# *Lycium barbarum* Polysaccharides Alleviate High‐Fat Diet–Induced Lipid Metabolism Disorder in *Takifugu obscurus*

**DOI:** 10.1155/anu/9289590

**Published:** 2026-04-29

**Authors:** Shenglin Yue, Fuqiang Wang, Zixiang Lin, Yiyang Huang, Tinghao Ma, Yingkang Sun, Yuzhe Han, Tongjun Ren, Xiaoran Zhao, Xiuli Wang

**Affiliations:** ^1^ Key Laboratory of Pufferfish Breeding and Culture in Liaoning Province, Dalian Ocean University, Dalian, 116023, China, dlou.edu.cn; ^2^ Key Laboratory of Applied Biology and Aquaculture of Northern Fishes in Liaoning Province, Dalian Ocean University, Dalian, 116023, China, dlou.edu.cn; ^3^ College of Fisheries and Life Sciences, Dalian Ocean University, Dalian, 116023, China, dlou.edu.cn

**Keywords:** gut–liver axis, lipid metabolism, *Lycium barbarum*, *Takifugu obscurus*

## Abstract

Excessive dietary fat intake disrupts lipid metabolism and induces oxidative stress and inflammation in aquatic species, impairing growth and health. In *Takifugu obscurus*, a widely cultivated fish species in China, fat predominantly accumulates in the liver, making this species particularly susceptible to hepatic lipid metabolism disorders. *Lycium barbarum* polysaccharides (LBPs), as a biologically active macromolecule, are capable of regulating lipid metabolism. Therefore, in this study, we aimed to investigate the regulatory effects of dietary LBP supplementation on lipid metabolism, hepatic function, intestinal microbiota, and metabolomic profiles in high‐fat diet (HFD)–fed *T. obscurus*. A control diet comprising 80 g/kg fat and five HFDs (150 g/kg fat) supplemented with 0, 0.5, 1.0, 1.5, and 2.0 g/kg LBP was administered to 630 juvenile *T. obscurus* individuals for 56 days. Serum biochemistry, hepatic antioxidant indices, gene expression, intestinal microbiota, and liver metabolomics were analyzed. HFD feeding significantly increased serum triglyceride (TG), total bile acid (TBA), total cholesterol (TCHO), aspartate aminotransferase (AST), alanine aminotransferase (ALT), and hepatic malondialdehyde (MDA) levels while decreasing high‐density lipoprotein cholesterol (HDL‐C) and total superoxide dismutase (T‐SOD) levels. Conversely, LBP supplementation significantly reversed these effects and improved the antioxidant capacity. LBP supplementation resulted in the downregulation of lipid synthesis and inflammatory gene expression and upregulation of lipid catabolism and antioxidant genes. Microbial and metabolomic analyses indicated that LBP supplementation restored gut microbial balance, enhanced diversity, and normalized bile acid and glycerophospholipid metabolism, exhibiting the most pronounced effects at a dose of 1.5 g/kg. In conclusion, LBP effectively mitigates HFD‐induced lipid metabolism disorders in *T. obscurus*. Furthermore, based on the strong correlations observed between the gut microbiota and differential metabolites, we speculate that the gut–liver axis may play a potential role in the regulatory effects of LBP, providing valuable clues for further investigation into the underlying molecular mechanisms.

## 1. Introduction

Aquaculture, the fastest‐growing sector in global animal production, has become a critical contributor to global food security [1]. However, rapid intensification has caused severe feed shortages, threatening industry sustainability. Therefore, improving nutrient utilization efficiency in fish is essential for sustainable development [2]. One widely adopted strategy involves increasing dietary lipid levels to reduce protein consumption and enhance protein utilization efficiency, an approach known as the “protein‐sparing effect” [3]. As an efficient energy source, dietary lipids—when supplemented in appropriate amounts—not only enhance growth performance but also supply fat‐soluble vitamins and support the structural integrity of phospholipids [4]. However, excessive lipid supplementation can lead to adverse outcomes, including reduced appetite [5], lipid metabolism disorders, liver damage, and suppressed immune function [6], ultimately reducing growth performance [7]. While research has attempted to address these issues using antibiotics [8], minimizing antibiotic use and developing eco‐friendly feed additives are vital for both human health and the long‐term sustainability of the aquaculture industry [9].

*T. obscurus*, a migratory fish species primarily distributed in the Yellow Sea and the East China Sea, is widely cultivated in China, owing to its rapid growth and high flesh quality [10]. This species exhibits a unique lipid storage pattern, with fat predominantly accumulating in the liver, whereas lipid deposition in the abdominal cavity and muscles is minimal [11]. This lipid storage pattern renders *T. obscurus* particularly susceptible to hepatic lipid metabolism disorders [12], underscoring the importance of investigating hepatic lipid metabolism in this species. Moreover, as a typical carnivorous species, *T. obscurus* requires high levels of dietary proteins and lipids, further increasing the risk of lipid metabolism disorders.

Despite their efficacy in treating fish lipid disorders, pharmaceuticals risk causing hepatic damage, gut dysbiosis, and residue accumulation [13, 14]. Consequently, natural extracts are gaining attention as eco‐friendly antibiotic alternatives due to their lower toxicity and reduced residue accumulation compared to synthetic compounds [15]. For example, dietary bile acids can alleviate lipid metabolism disorders in *Larimichthys crocea* [16] and *Oreochromis niloticus* [17], demonstrating that non‐nutritive additives can effectively improve lipid metabolic balance in fish.

*Lycium barbarum* (goji), a traditional medicinal herb that has been historically used across Asia, contains various bioactive compounds, such as polysaccharides, flavonoids, and carotenoids [18]. Among these, *L. barbarum* polysaccharides (LBPs) are the principal active constituents responsible for its health‐promoting effects [19, 20]. The antioxidant, immunomodulatory, and gut microbiota–modulating effects of LBP have been comprehensively summarized [21]. However, to date, research on polysaccharides in fish nutrition has mainly focused on their immunomodulatory and antioxidant functions [22, 23]. Notably, dietary supplementation with LBP can partially alleviate lipid metabolism disorders in *Cyprinus carpio* [24], *Epinephelus lanceolatus ♂* × *E. fuscoguttatus ♀* [25], and *Lateolabrax maculatus* [26]. However, differing lipid storage mechanisms among species may influence their responses to LBP. While previous research has focused primarily on gut microbiota, the impact of LBP on hepatic metabolites remains largely unclear.

Fish oil is a high‐quality lipid source in aquafeeds and constitutes a major component of dietary lipids. However, prolonged intake of high‐dose fish oil may induce hepatic lipid accumulation, reducing growth performance and feed conversion efficiency [27, 28]. The present study aimed to investigate the effects of LBP supplementation (0.5, 1.0, 1.5, and 2.0 g/kg) on the growth performance of *T. obscurus* fed a high‐fat diet (HFD) and explore its potential as a functional additive in aquafeed formulations. We analyzed the monosaccharide composition of LBP and integrated gut microbiota and metabolomic analyses to elucidate their mechanistic roles, offering new insights for future nutritional strategies. In this study, we aimed to enhance dietary lipid inclusion, reduce protein costs and nitrogen emissions, and mitigate lipid metabolism disorders in fish.

## 2. Materials and Methods

### 2.1. Animal Ethics Statement

This study was approved by the Animal Ethics Committee of Dalian Ocean University (Approval No: 202500009) and complied with the relevant institutional and ethical guidelines.

### 2.2. Determination of LBP Composition

LBPs were obtained as a commercial product (Catalog No. SP9311, Batch No. 2550614003) from Beijing Solarbio Science and Technology Co., Ltd. Their purity was verified by the supplier using ultraviolet spectrophotometry, with the results indicating ≥90% purity.

Polysaccharide purity was further validated using ultraviolet–visible spectrophotometry in the present study. The monosaccharide composition of LBP was analyzed using ion chromatography (ICS‐5000; Thermo Fisher Scientific, Waltham, MA, USA), as described by Ma et al. [29]. Briefly, LBP (5.0 mg) was placed in an ampoule containing 2 mL of 3 mol/L trifluoroacetic acid, purged with nitrogen, sealed, and hydrolyzed in an oil bath at 120°C for 3 h. After cooling to room temperature(25°C), residual acid was removed through nitrogen evaporation, and the hydrolysate was redissolved in 1 mL of ultrapure water. A 10 μL aliquot of this solution was diluted with 990 μL ultrapure water and centrifuged at 12,000 r/min for 5 min. The supernatant was then filtered through a 0.22 μm aqueous phase filter membrane before analysis.

Ion chromatography was performed using a Dionex ICS‐5000 ion chromatography system (Thermo Fisher Scientific) equipped with a CarboPac PA20 anion‐exchange column (3 mm × 150 mm; Thermo Fisher Scientific), and a pulsed amperometric detector was used. The column temperature was maintained at 30°C, with a flow rate of 0.3 mL/min and an injection volume of 25 μL. The mobile phases were as follows: A, ultrapure water; B, 15 mM NaOH; and C, 15 mM NaOH and 100 mM NaOAc. Gradient elution was programmed as follows: 0–18 min 98.8% A and 1.2% B, 20–30 min 50% A and 50% B, 30.1–46 min 100% C, 46.1–50 min 100% B, and 50.1–80 min, returning to 98.8% A and 1.2% B.

The molecular weights and conformations of the polysaccharides were determined using high‐performance size exclusion chromatography equipped with multiple‐angle laser light‐scattering and refractive index. LBP (10.0 mg) was dissolved in ultrapure water (1.0 mL; 10.0 mg·mL^−1^) and filtered through a 0.22 μm membrane before injection. Analysis was performed using an Agilent 1260 high‐performance liquid chromatography (HPLC) system (Agilent Technologies, Santa Clara, CA, USA) coupled with a Shodex OHpak SB‐806 HQ/SB‐804 HQ column (7.9 × 300 mm; Shodex, Tokyo, Japan). The mobile phase consisted of 0.2 M NaCl at a flow rate of 1.0 mL·min^−1^. Detection was carried out using a Wyatt DAWN laser light‐scattering instrument (Wyatt Technology, Santa Barbara, CA, USA) and an Agilent RID‐G7162A refractive index detector (Agilent Technologies). Molecular weight calculations were performed using ASTRA 8 software (Wyatt Technology).

### 2.3. Experimental Diet

Two control diets were prepared: a normal‐fat diet (NFD) and an HFD. HFD was formulated by increasing the amount of fish oil to induce lipid metabolism disorders, while wheat flour was adjusted to balance the formulation. Based on a previously described method [30], the HFD was supplemented with five concentrations of LBP (0,0.5, 1.0, 1.5, and 2.0 g/kg) to formulate five experimental diets, designated HFD, HFD1, HFD2, HFD3, and HFD4, respectively. Table 1 presents the composition of the basal diet.

**Table 1:** Ingredients and formulation of the basal diet (g/kg).

Item	NFD	HFD	HFD1	HFD2	HFD3	HFD4
Fish meal	500	500	500	500	500	500
Soybean meal	160	160	160	160	160	160
Wheat flour	215	145	145	145	145	145
Carboxymethyl cellulose	20	20	19.5	19	18.5	18
LBP^a^	0	0	0.5	1	1.5	2
Fish oil	40	110	110	110	110	110
Choline (50%)	5	5	5	5	5	5
Multivitamin^b^	10	10	10	10	10	10
Compound minerals^c^	10	10	10	10	10	10
Yeast	40	40	40	40	40	40
Total	1000	1000	1000	1000	1000	1000
Nutrient level (g/kg)						
Moisture	94.211	95.513	96.825	100.341	96.276	98.627
Crude protein	451.019	448.584	449.328	448.559	447.101	449.839
Crude fat	81.594	152.297	158.511	152.312	153.067	153.926

^a^ *Lycium barbarum* polysaccharides (LBPs) (purity ≥ 90%) were purchased from Beijing Solarbio Science & Technology Co., Ltd.

^b^The multivitamin premix (provided by Guangzhou Southern Biotechnology Co., Ltd.) contained the following per kilogram: vitamin B1, 10,000 mg; vitamin B2, 10,000 mg; vitamin B6, 10,000 mg; and anhydrous glucose as the carrier (moisture content < 10%).

^c^The mineral premix (provided by Anhui Zhuokai Biotechnology Co., Ltd.) contained the following per kilogram: Cu, 6000–10,000 mg; Fe, 30,000–50,000 mg; Zn, 6000–10,000 mg; Mn, 15,000–25,000 mg; and Mg, 8000–13,000 mg.

All ingredients were ground using a pulverizer, passed through an 80‐mesh sieve, and mixed thoroughly via stepwise blending. Water (250 mL) was added to adjust the moisture content, and the mixture was pelleted (2–2.5 mm diameter) using a feed pelletizer (Pinzheng Equipment Co., Ltd., China). Subsequently, the pellets were dried in hot air at 55°C for 8 h, cooled to room temperature, sealed, and stored at –20°C until use.

### 2.4. Experimental Fish and Husbandry Management

Animal experiments were conducted at the Aquatic Animal Nutrition and Feed Laboratory, Dalian Ocean University, Liaoning Province, China. Experimental fish were obtained from Shuangyang Seed Industry Technology (Nantong) Co., Ltd., Jiangsu, China. Before the start of the experiment, the rearing water salinity was adjusted to 4 ppt using marine salt (Qingdao Hai Zhi Yan Aquarium Technology Co., Ltd., China), following the protocol described by Kato et al. [31].

Fish were placed in 1500 L tanks and initially fasted for 3 days, followed by a 1‐week acclimation period during which they were fed the control diet to ensure adaptation to the rearing environment. At the start of the experiment, 630 healthy and size‐uniform juvenile *T. obscurus* (3.167 ± 0.102 g) were selected. After a 24‐h fasting period, the fish were randomly divided into six groups, designated as NFD, HFD, HFD1, HFD2, HFD3, and HFD4, with 35 fish per tank and three replicates per group, totaling 18 groups. The experiment lasted for 56 days.

A recirculating aquaculture system was used throughout the trial, with daily replacement of filter cotton and continuous aeration. During the experimental period, fish were fed at a daily rate of 3%–4% of their body weight, with feedings at 09:00, 13:00, and 18:00 h. Every 14 days, three fish were randomly sampled from each tank for weighing, and feeding amounts were adjusted accordingly. Water with a salinity of 4 ppt was exchanged twice daily at 10:30 h and 19:30 h. After each water exchange, salinity was measured using a salinometer to ensure it was consistently maintained at ~4 ppt. Water quality parameters were maintained as follows: temperature of 24–26°C, pH of ~7.7, dissolved oxygen above 6.0 mg/L, nitrite concentration below 0.01 mg/L, and ammonia nitrogen concentration below 0.20 mg/L.

### 2.5. Sample Collection

At the end of the trial, fish were fasted for 24 h, the recirculating water system was shut down, and *T. obscurus* was anesthetized using MS‐222. Six fish were randomly selected from each tank to assess growth performance and related parameters, including body length, body weight, liver weight, and visceral weight. Livers were preserved in 4% paraformaldehyde for histological analysis, including hematoxylin‐eosin (H&E) and Oil Red O staining.

Blood samples were obtained via the caudal vein of 10 randomly selected fish per tank using sterile syringes prerinsed with heparin sodium. Blood samples were centrifuged at 3500 rpm for 10 min at low temperature to separate the serum, which was then immediately flash‐frozen in liquid nitrogen and stored at −80°C. Enzymatic activity assays were performed within 24 h.

Three additional fish were randomly selected, and their liver and intestinal tissues were collected, rapidly frozen in liquid nitrogen, and stored at −80°C for enzymatic activity analysis within 24 h. Three fish were also randomly selected from each group for intestinal tissue collection to assess gut microbial composition. The tissues were placed in centrifuge tubes, flash‐frozen in liquid nitrogen, and stored at −80°C. Finally, nine fish were randomly selected for liver tissue sampling and stored in sterile microcentrifuge tubes for analysis of genes related to lipid metabolism. All procedures were performed in sterile, ice‐cold trays to ensure sample integrity.

### 2.6. Growth Performance Evaluation

Body weight, body length, and liver weight data were used to calculate the following growth performance indices:

Survival rate SR,% = Final number of fish /Initial number of fish×100,

Weight gain rate WGR,%=Final body weight −Initial body weight /Initial body weight×100,

Specific growth rate SGR,%/day=Ln final body weight −Ln initial body weight /Rearing days×100,

Feed conversion ratio FCR =Average feed intake / Final body weight −Initial body weight,

Condition factor CF,%=Final body weight / Final body length3×100,

Hepatosomatic index HSI,%=Liver weight /Final body weight×100,

Viscerosomatic index VSI,%=Viscera weight /Final body weight×100.

### 2.7. Diet Formulation and Proximate Analysis

Feed and whole‐body compositions were analyzed according to the methods of the Association of Official Analytical Chemists [32]. Crude protein content was determined using the Kjeldahl method (Kjeltec KDN‐1000 Auto Analyser, Shanghai Xinrui Instrument and Meter Co., Ltd., Shanghai, China). Crude lipid content was quantified via ether extraction using a Soxhlet apparatus (QW‐SZF‐06A; Hangzhou Qiwei Instrument Co., Ltd., Hangzhou, China). Ash content was determined by ashing the samples at 550°C for 8 h in a muffle furnace (Shanghai Yuejin Medical Instrument Co., Ltd., Shanghai, China). Moisture content was determined by drying the samples to a constant weight in a thermostatic oven at 105°C.

### 2.8. Serum Biochemistry and Hepatic Oxidative Stress Assays

Enzymatic activity was assessed using commercial assay kits in serum, liver, and intestinal samples following the manufacturer’s instructions. Serum biochemical parameters included total cholesterol (TCHO), triglyceride (TG), high‐density lipoprotein cholesterol (HDL‐C), low‐density lipoprotein cholesterol (LDL‐C), total protein (TP), total bile acid (TBA), aspartate aminotransferase (AST), and alanine aminotransferase (ALT) (catalog numbers A111‐1‐1, A110‐1‐1, A112‐1‐1, A113‐1‐1, A154‐1‐1, A045‐2‐2, E003‐2‐1, C009‐2‐1, and C010‐2‐1, respectively; Nanjing Jiancheng Bioengineering Institute, Nanjing, China). The following liver enzymes were assessed: total superoxide dismutase (T‐SOD), TP, and malondialdehyde (MDA) (kit catalog numbers A001‐3‐2, A045‐2‐2, and A003‐1‐2, respectively; Nanjing Jiancheng Bioengineering Institute). All measurements were performed using a microplate reader (ReadMax 500 F, Shanghai Shanpu Biotechnology Co., Ltd., Shanghai, China).

### 2.9. Histological Analysis and Oil Red O Staining of Liver Tissue

Histological analysis of the liver was performed using H&E staining. Liver samples were fixed in 4% paraformaldehyde for 24 h, dehydrated using a graded ethanol series (70%, 80%, 90%, 95%, and 100%), cleared in xylene, and embedded in paraffin. Sections were prepared using a microtome (RM2016; Leica, Wetzlar, Germany), transferred to 50°C water, and subsequently dried, deparaffinized, and rehydrated. Staining was performed with H&E, and tissue morphology was examined under a light microscope (Nikon Eclipse E100; Nikon, Tokyo, Japan). Images were captured using CaseViewer software (Digital Pathology Association, Carmel, IN, USA).

Liver tissues were sectioned to a thickness of 8 μm using a cryostat (CRYOSTAR NX50; Thermo Fisher Scientific), fixed with formaldehyde, and treated with isopropanol. To visualize hepatic lipid accumulation, sections were stained with Oil Red O and hematoxylin, resulting in lipid droplets and nuclei (Nu) staining red and light blue, respectively. Following the method described by Mehlem et al. [33], five regions (upper, lower, left, right, and center) were selected from each liver section at 40× magnification using the CaseViewer Ver 2.2 software (Thermo Fisher Scientific). The relative area of lipid droplet accumulation was quantified using ImageJ software (National Institutes of Health, Bethesda, MD, USA).

### 2.10. RNA Extraction and Gene Expression Analysis

Total RNA was extracted from the liver samples of *T. obscurus* using a Total RNA Extraction Kit (Tiangen Biotech, Beijing, China). The concentration and purity of the isolated RNA were assessed using an Ultrospec 3100 Pro spectrophotometer (Amersham Bioscience, Amersham, UK), ensuring that the A260/A280 ratio remained between 1.8 and 2.0. Reverse transcription was performed using FastKing gDNA Dispelling RT SuperMix (Tiangen Biotech) to synthesize complementary cDNA from the purified RNA. The reaction was carried out using a QuantReady K9600 thermal cycler (Hangzhou Suizhen Biotechnology Co., Ltd., Hangzhou, China).

PCR amplification was performed in a 20 μL reaction volume using the following qPCR cycling conditions: an initial denaturation at 95°C for 2 min followed by 40 cycles of 95°C for 5 s and 60°C for 15 s, following the manufacturer’s instructions. Each sample was analyzed in triplicate. The relative expression levels of target genes were calculated using the 2^–ΔΔCT^ method, as described by Livak and Schmittgen [34]. The sequences of the primers used are listed in Table 2.

**Table 2:** Sequences of gene‐specific primers used for quantitative PCR.

Gene	Primer sequence (5′–3′)	Source
*β-Actin*	F: CATCACCATCGGCAACGAGAGG	[35]
	R: CGTCGCACTTCATGATGCTGTTG	
*Fas*	F: CAAGGAAGGCATTATGGGAGG	[35]
	R: TGGAAAAGACACAGGGGCAG	
*Me*	F: GCCATCGGTGAGGGCATAG	[35]
	R: GCCTGAACGAACGACCAATC	
*G6pd*	F: ATTGATAAACTGGTTCCTCTACTCG	[35]
	R: CTTCTTCTCCGCCACTGACTC	
*Dgat1*	F: GCTACAGCAACTACAGGGGG	[35]
	R: AGACACGACTTGGATGGGG	
*Cpt1*	F: TTCCAGTTCACCGTCTCCC	[35]
	R: CAGCAGTCATCACCCCATTC	
*Ppar-γ*	F: GAAAGGCGTTAAACAGAAGCAA	[35]
	R: GGGTGGAGGAAGATGAGATGG	
*Ppar-α*	F: TCTCAAATGTCTGTCTGTGGG	[35]
	R: AGTTTTTGATGTAGGCGTCG	
*AMPK*	F: GCTCTGGAGTTCGGACTTTTG	[35]
	R: GCATTTCTTACTGGGGTGACG	
*TNF-α*	F: TCGTGGTGGTCCTCTGTTGC	[36]
	R: CTTGGCTTTGCTGCTGATGC	
*Cat*	F: TGAGCCAAGCCCTGACAAGATGC	[37]
	R: GGTAGTTGGCCACACGGGTTCTG	
*Mn-Sod*	F: AGATGTCCGCCGCTACAGTTGC	[37]
	R: GCCAAGGAGCGGGATGAGACC	
*IL-6*	F: GCTGGAAAACAAGGTGAGGGA	[38]
	R: TGTGGAAGGTGTCGGGGTAGT	

### 2.11. Intestinal Microbial Profiling

Following a 24‐h fasting period at the end of the experiment, three *T. obscurus* individuals were randomly selected from three parallel tanks in each treatment group and anesthetized. Dissection was performed using instruments sterilized with 75% ethanol to isolate the intestinal tissues. The collected samples were flash‐frozen in liquid nitrogen and stored at –80°C for subsequent gut microbial analysis, performed within 24 h.

Genomic DNA was extracted using an E.Z.N.A. DNA/RNA Isolation Kit (Omega Bio‐Tek, Norcross, GA, USA). DNA integrity was assessed using 1% (w/v) agarose gel electrophoresis, and concentration and purity were determined using a NanoDrop 2000 spectrophotometer (Thermo Fisher Scientific). PCR amplification of the target fragments was performed using an ABI GeneAmp 9700 thermal cycler (Applied Biosystems, Foster City, CA, USA) with specific primers targeting the V3–V4 hypervariable regions. PCR products were recovered using 2% agarose gel electrophoresis and purified using a DNA Gel Extraction and Purification Kit (PCR Clean‐Up Kit, Yuehua, China). The purified amplicons were pooled at equimolar concentrations and subjected to paired‐end sequencing using the BGI G99 platform.

Raw FASTQ files were processed using QIIME2 for dereplication and quality filtering. Sequences were clustered into operational taxonomic units (OTUs) at 97% similarity, and chimeric sequences were removed. Taxonomic classification was performed using the Silva database with a confidence threshold of 0.8. The primer sequences used for this sequencing were as follows: forward primer (CCTAYGGGRBGCASCAG) and reverse primer (GGACTACNNGGGTWTCTAAT).

### 2.12. Hepatic Metabolomic Analysis

#### 2.12.1. Liver Metabolite Extraction and Liquid Chromatography (LC)‐Mass Spectrometry (MS) Analysis

Metabolites were extracted from the liver tissues of *T. obscurus* individuals. Samples stored at –80°C were thawed on ice. After cryogenic grinding in liquid nitrogen, 400 μL of methanol:water (7 : 3, v/v; Millipore Sigma, Burlington, MA, USA) was added to 20 mg of homogenized tissue. The mixture was vortexed at 1500 rpm for 5 min (Eppendorf, Hamburg, Germany) and left on ice for 15 min. Samples were then centrifuged at 12,000 rpm for 10 min at 4°C. A 300 μL aliquot of supernatant was collected, frozen at –20°C for 30 min, and centrifuged again at 12,000 rpm for 3 min at 4°C. Finally, 200 μL of the clarified supernatant was collected for LC–MS analysis.

Metabolites were analyzed using HPLC (Waters ACQUITY Premier HSS T3 Column 1.8 µm; Waters Corp., Milford, MA, USA). Samples were divided into two portions for analysis: one analyzed in positive ion mode and one in negative ion mode, using the same elution gradient. In positive mode, solvent A (0.1% formic acid in water) and solvent B (0.1% formic acid in acetonitrile) were used. The gradient elution program was as follows: 5% to 20% B over 1 min, increased to 99% B in 2 min, held for 1.5 min, returned to 5% B in 0.1 min, and maintained for 1.4 min. Column temperature was maintained at 40°C, with a flow rate of 0.4 mL/min and an injection volume of 4 μL.

Full‐scan MS were acquired by alternating full MS and data‐dependent MS/MS scans over an m/z range of 75–1000 at a resolution of 35,000. The spray voltage was set to 3500 V in positive ion mode and 3200 V in negative ion mode. The sheath gas flow rate (Arb) was set to 30, with an auxiliary gas flow rate of five. The ion transfer tube and vaporizer temperatures were maintained at 320°C and 300°C, respectively. Collision energies were set to 30, 40, and 50 V. The intensity threshold was defined as 1.00 × 10^6^ cps. The top N acquisition was configured with *N* = 10 and a dynamic exclusion duration of 3 s.

#### 2.12.2. Hepatic Metabolomic Data Processing and Statistical Analysis

LC‐MS data were analyzed using Profinder software (Agilent Technologies) to obtain the retention time, peak area, and related information. The resulting data files were then imported into Mass Profiler Professional (Agilent Technologies; Strand Life Sciences, Bengaluru, India) in CEF format for peak alignment and statistical analysis. Compound classification and metabolic pathway annotation were performed using the Kyoto Encyclopedia of Genes and Genomes (KEGG) database. Pairwise comparisons were conducted using Student’s *t*‐test. After false discovery rate correction, differential metabolites were identified based on the following criteria: variable importance in projection (VIP) > 1, false discovery rate < 0.05, and fold change ≥ 3 (upregulated) or ≤ 0.333 (downregulated).

Prior to orthogonal partial least squares discriminant analysis (OPLS‐DA), data were log‐transformed and mean‐centered. VIP scores were extracted from the OPLS‐DA model, and a 200‐permutation test was performed to prevent model overfitting.

### 2.13. Statistical Analysis

Growth‐related parameters are expressed as mean ± standard deviation (mean ± SD). Statistical analyses were performed using IBM SPSS Statistics 29 software (IBM Corp., Armonk, NY, USA). Independent‐sample *t*‐tests were used to compare the HFD and NFD groups. For comparisons among the LBP intervention groups (HFD1, HFD2, HFD3, and HFD4) and the two control groups, data were first tested for normality and homogeneity of variance. Upon satisfying these assumptions, one‐way analysis of variance was conducted, followed by intergroup comparisons using the Waller–Duncan post hoc test. In addition, for *α*‐diversity analysis, the Pielou, Simpson, and Shannon indices were calculated using the “vegan” package in R v4.0.2.

## 3. Results

### 3.1. Analysis of LBP Composition

Using a mixture of 15 monosaccharides (fucose, galactosamine, rhamnose, arabinose, glucosamine, galactose, glucose, xylose, mannose, fructose, ribose, galacturonic acid, guluronic acid, glucuronic acid, and mannuronic acid) as external standards, we calculated the molar ratios and contents (µg/mg) of each monosaccharide in the samples based on chromatographic peak areas. Supporting Information 1: Table S1 lists the sources, peak areas, retention times, and purities of these 15 monosaccharide standards. Supporting Information 2: Figure S1A depicts their total ion chromatograms (TICs), while Supporting Information 2: Figure S1B displays the monosaccharide components detected in the LBP. Table 3 presents the monosaccharide content of LBP. The signal peak at 2.0 min corresponded to the solvent peak of sodium hydroxide, while the chromatographic peak at 40 min represented the sodium acetate signal. The system peak appeared at 35 min, while the peak preceding fucose indicated fructose degradation. The molecular weight of LBP was 5.094 × 10^4^ Da (±16.417%) (Supporting Information 3: Figure S2). The molecular conformation diagram of LBP is presented in Supporting Information 4: Figure S3.

**Table 3:** Monosaccharide content in *Lycium barbarum* polysaccharides.

Compounds	Peak area	Retention time (min)	Molar ratio	Concentration (μg/mg)
Arabinose	0.129	11.95	0.002	0.757
Galactose	0.255	15.575	0.005	2.077
Glucose	97.153	17.484	0.796	325.523
Mannose	13.119	21.85	0.188	77.082
Galacturonic acid	0.336	42.775	0.006	2.734
Glucuronic acid	0.226	45.134	0.002	0.96

### 3.2. Growth Performance and Body Composition of *T. obscurus*

The effects of different LBP supplementation levels on the growth performance and feed utilization of *T. obscurus* are presented in Table 4. We observed no significant differences in condition factor (CF) or survival rate (SR) among the different treatment groups (*p* > 0.05). In the HFD group, the final body weight (FBW), weight gain rate (WGR), and specific growth rate (SGR) of *T. obscurus* individuals were significantly reduced compared to the NFD group (*p* < 0.05). However, these reductions were reversed by supplementation with 0.5 or 1.0 g/kg LBP (*p* < 0.05). Notably, under HFD conditions, supplementation with 0.5 g/kg LBP significantly increased the FBW, WGR, and SGR compared to the NFD group (*p* < 0.05). In addition, the HSI and viscerosomatic index (VSI) of *T. obscurus* individuals were significantly increased (*p* < 0.05) in the HFD group compared to those in the NFD group, whereas supplementation with 0.5, 1.0, or 1.5 g/kg LBP significantly reduced both indices. The results indicated that a HFD reduced the growth rate of the fish and increased both the HSI and VSI, whereas supplementation with 0.5–2 g/kg LBP significantly mitigated these adverse effects.

**Table 4:** Effect of *Lycium barbarum* polysaccharides on indices related to growth performance of *T. obscurus* individuals receiving a high‐fat diet (HFD).

Item	NFD	HFD	HFD1	HFD2	HFD3	HFD4
IBW (g)	3.125 ± 0.093	3.19 ± 0.093	3.155 ± 0.124	3.149 ± 0.067	3.191 ± 0.09	3.193 ± 0.128
FBW (g)	9.37 ± 0.228^d^	8.877 ± 0.371^e^	9.679 ± 0.385^c^	10.111 ± 0.482^b^	10.683 ± 0.365^a^	10.528 ± 0.534^a^
WGR (%)	197.621 ± 8.767^d^	178.456 ± 13.507^e^	207.05 ± 12.255^c^	221.833 ± 14.154^b^	234.9 ± 11.243^a^	229.769 ± 12.33^a^
SGR (% day^−1^)	1.947 ± 0.054^d^	1.827 ± 0.088^e^	2.002 ± 0.071^c^	2.086 ± 0.079^b^	2.157 ± 0.059^a^	2.13 ± 0.067^a^
VSI (%)	11.27 ± 0.639^d^	13.881 ± 0.704^a^	12.625 ± 0.679^b^	12.488 ± 0.61^b^	11.822 ± 0.577^c^	11.375 ± 0.595^d^
HSI (%)	7.667 ± 0.659^d^	10.15 ± 0.81^a^	8.994 ± 0.864^b^	8.525 ± 0.76^b^	8.243 ± 0.857^c^	7.68 ± 0.861^d^
FCR (%)	1.974 ± 0.047^b^	2.085 ± 0.088^a^	1.93 ± 0.055^c^	1.867 ± 0.057^d^	1.816 ± 0.039^e^	1.835 ± 0.047^de^
CF (g/cm^3^)	3.304 ± 0.657	3.403 ± 0.771	3.268 ± 0.551	3.305 ± 0.657	3.274 ± 0.876	3.467 ± 0.451
SR (%)	86.667 ± 6.598	79.048 ± 1.65	83.81 ± 4.364	82.857 ± 5.714	82.857 ± 5.714	82.857 ± 4.949

*Note:* Values represent the means ± SD of three replicates. Means in the same row with different superscripts are significantly different (*p* < 0.05).

Abbreviations: CF, condition factor; FBW, final body weight; FCR, feed conversion ratio; HIS, hepatosomatic index; IBW, initial body weight; NFD, normal‐fat diet; SGR, specific growth rate; SR, survival rate; VSI, viscerosomatic index; WGR, weight gain rate.

The whole‐body composition of *T. obscurus* individuals in the different LBP groups is presented in Table 5. We observed no significant differences in muscle moisture or crude protein content among the treatment groups. However, compared with the NFD group, crude lipid levels were significantly increased in both the whole body and liver of *T. obscurus* individuals in the HFD group (*p* < 0.05), whereas they were markedly reduced following LBP supplementation. In addition, the HFD significantly increased whole‐body crude protein content (*p* < 0.05) and significantly decreased liver moisture content (*p* < 0.05). The results showed that a HFD significantly increased crude lipid levels in both the liver and whole body while having no significant effect on muscle crude lipid content. Supplementation with LBP markedly reduced lipid accumulation in the liver and whole body.

**Table 5:** Effects of *Lycium barbarum* polysaccharides on body composition‐related indicators of *T. obscurus* fed a high‐fat diet (HFD).

Item	NFD	HFD	HFD1	HFD2	HFD3	HFD4
Whole body						
Moisture (%)	69.965 ± 0.565	70.408 ± 0.857	70.031 ± 0.742	71.471 ± 0.932	71.139 ± 0.509	70.86 ± 0.684
Crude protein (%)	11.933 ± 0.321^b^	12.666 ± 0.428^a^	13.015 ± 0.224^a^	12.947 ± 0.336^a^	12.942 ± 0.185^a^	12.936 ± 0.448^a^
Crude lipid (%)	10.864 ± 0.798^e^	17.286 ± 0.691^a^	15.56 ± 0.898^b^	14.132 ± 0.823^c^	13.712 ± 0.666^d^	14.087 ± 0.599^d^
Liver						
Moisture (%)	24.766 ± 0.835^a^	20.478 ± 0.5^cd^	20.113 ± 0.59^e^	21.204 ± 0.326^c^	22.523 ± 0.576^b^	22.467 ± 0.848^b^
Crude lipid (%)	50.031 ± 0.711^e^	73.064 ± 0.819^a^	69.354 ± 0.879^b^	60.937 ± 0.563^c^	58.367 ± 0.887^d^	58.011 ± 0.734^d^
Muscle						
Moisture (%)	77.524 ± 0.69	77.663 ± 0.261	77.979 ± 0.276	77.169 ± 0.854	77.647 ± 0.311	78.598 ± 0.884
Crude protein (%)	18.555 ± 0.367	19.838 ± 1.478	18.629 ± 0.773	19.369 ± 0.444	19.885 ± 0.559	19.037 ± 0.554

*Note:* Values represent mean ± SD of triplicate experiments. Differences in the mean values of different superscripts in the same original data were statistically significant (*p* < 0.05).

### 3.3. Analysis of Serum and Hepatic Enzyme Activities

The effects of different LBP supplementation levels on the serum and hepatic enzyme activities of HFD‐fed *T. obscurus* individuals are summarized in Table 6. The HFD group exhibited higher serum TCHO, TG, TBA, LDL‐C, AST, and ALT levels than the NFD group (*p* < 0.05). In contrast, the serum levels of TCHO, TG, AST, ALT, and TBA were significantly reduced in all LBP supplementation groups (*p* < 0.05) compared to those in the HFD group. Although LDL‐C levels in the HFD1 and HFD2 groups exhibited a decreasing trend, these differences were not statistically significant (*p* > 0.05). However, serum LDL‐C levels in the HFD3 and HFD4 groups were significantly lower than those in the HFD group (*p* < 0.05).

**Table 6:** Effects of *Lycium barbarum* polysaccharides on serum and hepatic enzyme activities of *T. obscurus* fed a high‐fat diet (HFD).

Item	NFD	HFD	HFD1	HFD2	HFD3	HFD4
Serum						
LDL‐C (mmol/L)	1.378 ± 0.182^c^	2.853 ± 0.421^a^	2.692 ± 0.232^a^	2.621 ± 0.275^a^	2.061 ± 0.154^b^	2.015 ± 0.337^b^
HDL‐C (mmol/L)	1.832 ± 0.128^a^	1.208 ± 0.085^d^	1.26 ± 0.078^d^	1.277 ± 0.217^cd^	1.515 ± 0.163^bc^	1.536 ± 0.116^b^
TG (mmol/L)	1.612 ± 0.071^c^	2.138 ± 0.076^a^	1.852 ± 0.048	1.741 ± 0.079^bc^	1.618 ± 0.16^c^	1.705 ± 0.054^bc^
TCHO (mmol/L)	2.791 ± 0.183^d^	5.054 ± 0.222^a^	4.146 ± 0.1^cb^	4.431 ± 0.171^b^	3.655 ± 0.141^c^	3.732 ± 0.305^c^
TBA (µmol/L)	20.389 ± 1.989^d^	43.687 ± 4.858^a^	30.834 ± 1.297^b^	27.795 ± 4.796^bc^	22.468 ± 4.574^cd^	23.807 ± 2.471^cd^
ALT (U/L)	7.351 ± 0.968^c^	17.067 ± 1.351^a^	13.411 ± 2.339^b^	9.514 ± 0.515^c^	8.085 ± 2.134^c^	9.048 ± 1.789^c^
AST (U/L)	12.127 ± 2.091^c^	25.193 ± 5.569^a^	20.053 ± 3.673^ab^	19.939 ± 2.244^ab^	18.363 ± 1.088^b^	19.05 ± 2.553^b^
Liver						
T‐SOD (U/mgprot)	161.059 ± 7.147^a^	106.018 ± 6.696^d^	125.239 ± 9.677^c^	154.164 ± 10.669^ab^	146.483 ± 8.233^b^	130.932 ± 7.08^c^
MDA (U/gprot)	5.401 ± 1.33^c^	9.488 ± 0.752^a^	7.394 ± 0.707^b^	6.774 ± 1.026^bc^	5.846 ± 0.278^c^	6.426 ± 0.755^bc^

*Note:* Values represent mean ± SD of triplicate experiments. Differences in the mean values of different superscripts in the same original data were statistically significant (*p* < 0.05).

Abbreviations: ALT, alanine aminotransferase; AST, aspartate aminotransferase; HDL‐C, high‐density lipoprotein cholesterol; LDL‐C, low‐density lipoprotein cholesterol; MDA, malondialdehyde; T‐SOD, total superoxide dismutase; TBA, total bile acid; TCHO, total cholesterol; TG, triglycerides.

The HFD group also exhibited significantly decreased serum HDL‐C levels (*p* < 0.05), whereas they were significantly increased in the HFD3 and HFD4 groups (*p* < 0.05). Notably, hepatic T‐SOD activity was significantly reduced in the HFD group (*p* < 0.05) but was restored by LBP supplementation (0.5–2.0 g/kg), with the highest activity observed in the HFD2 group (*p* < 0.05). In addition, hepatic MDA levels were significantly elevated in the HFD group compared to the NFD group; however, supplementation with LBP, at different percentages, significantly decreased these levels (*p* < 0.05).

### 3.4. Analysis of Liver Histology and Oil Red O Staining

**Figure 1:**
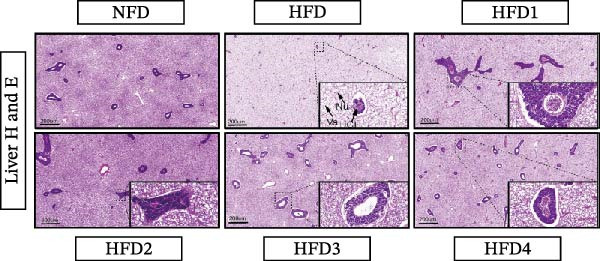
LBP on liver histopathology of *T. obscurus* under high‐fat diet. Note: The abbreviations in the figure are as follows: Nu, nuclei; Va, vacuolation; ICI, inflammatory cell infiltration. The figure displays liver histology at 5× magnification, with an inset in the lower right corner showing 40× magnification (scale bar: 200 μm).

As illustrated in Figure 1, an HFD induced marked histological alterations in the livers of *T. obscurus* individuals, characterized by extensive hepatic lipid vacuolization and mild infiltration of inflammatory cells. However, the degree of vacuolization was notably reduced following LBP supplementation at all concentrations, and the infiltration of inflammatory cells was markedly reduced in the HFD3 and HFD4 groups.

As depicted in Figure 2, hepatic lipid droplet deposition in *T. obscurus* individuals was significantly increased in the HFD group compared to that in the NFD group (*p* < 0.05). Notably, LBP supplementation (HFD1–HFD4) significantly alleviated hepatic lipid accumulation compared to that observed in the HFD group (*p* < 0.05). However, lipid levels in all LBP‐supplemented groups remained significantly higher than those in the NFD group (*p* < 0.05). In summary, an HFD induced marked hepatic steatosis in *T. obscurus*, whereas LBP supplementation partially but significantly alleviated these hepatic alterations.

Figure 2LBP staining of Oil Red O in the liver of *T. obscurus* under a high‐fat diet. (A) The figure shows the accumulation of lipid droplets in the liver at a magnification of 5×, and the lower right corner shows the accumulation of lipid droplets at a magnification of 40×. (B) The area occupied by five randomly selected regions per section in the field of vision at a magnification of 40×.

**Figure:**
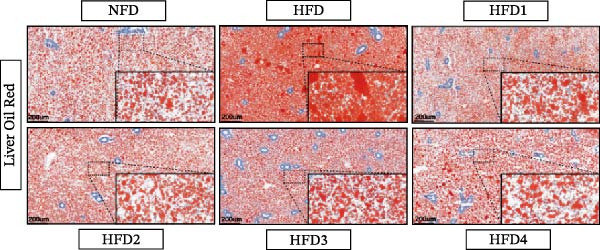
(A)

**Figure:**
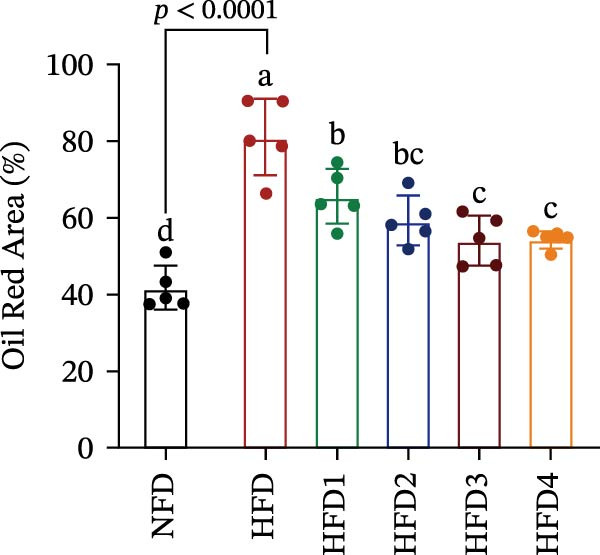
(B)

### 3.5. Analysis of Lipid Metabolism, Synthesis, and Expression of Inflammation‐Related Genes

The expression levels of genes associated with lipid metabolism, lipid synthesis, and inflammation are presented in Figure 3. We noted no significant differences in *Me* expression among the treatment groups (*p* > 0.05). Under HFD conditions, the expression of lipid metabolism‐related genes (*AMPK*, *Cpt1*, and *Ppar-α*) was significantly downregulated (*p* < 0.05); however, LBP supplementation significantly upregulated the expression of these genes across all treatment groups (*p* < 0.05). In addition, the expression of lipid synthesis‐related genes (*Ppar-γ*, *Fas*, *G6pd*, and *Dgat*) was significantly upregulated in the HFD group (*p* < 0.05). Following LBP supplementation, the expression levels of *Ppar-γ*, *Fas*, and *G6pd* were significantly downregulated in the HFD2, HFD3, and HFD4 groups (*p* < 0.05), whereas *Dgat* expression remained unchanged (*p* > 0.05). Notably, the expression levels of the proinflammatory cytokines *IL-6* and *TNF-α* were significantly upregulated in the HFD group (*p* < 0.05), and this effect was significantly reversed by LBP supplementation (*p* < 0.05). Moreover, the expression levels of the antioxidant genes *Cat* and *Mn-Sod* were downregulated in the HFD group (*p* < 0.05), whereas it was significantly increased following LBP supplementation (*p* < 0.05).

Figure 3Effects of LBP on lipid metabolism, synthesis, and inflammatory gene expression in the liver under a high‐fat diet. (A–L) Bar charts showing the expression levels of genes involved in lipid metabolism, inflammation, and antioxidant defense.

**Figure:**
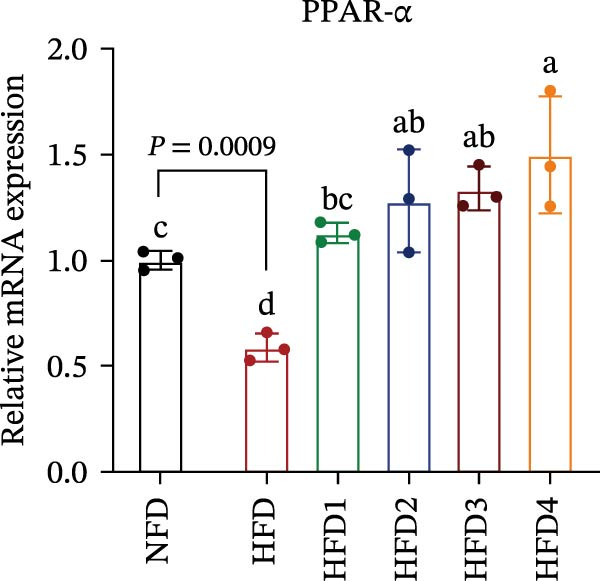
(A)

**Figure:**
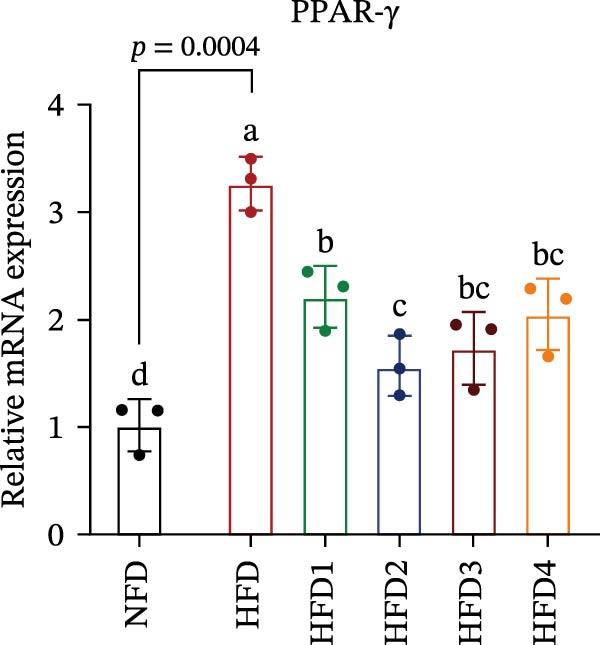
(B)

**Figure:**
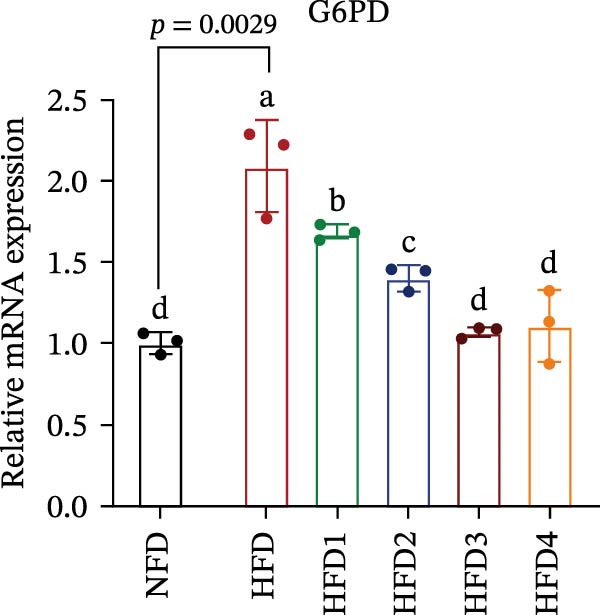
(C)

**Figure:**
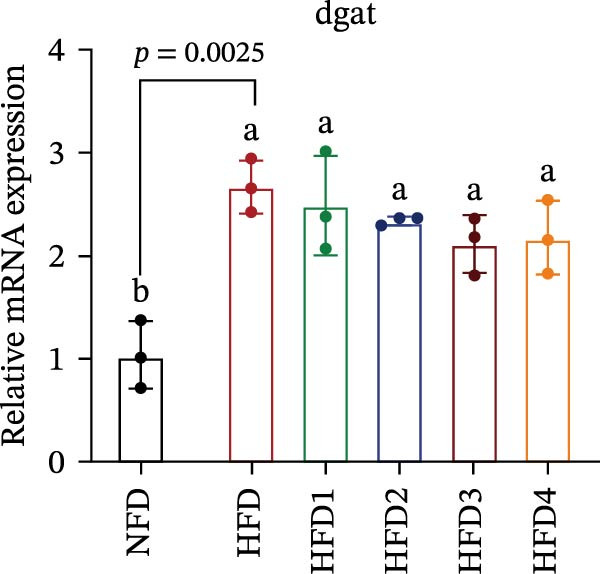
(D)

**Figure:**
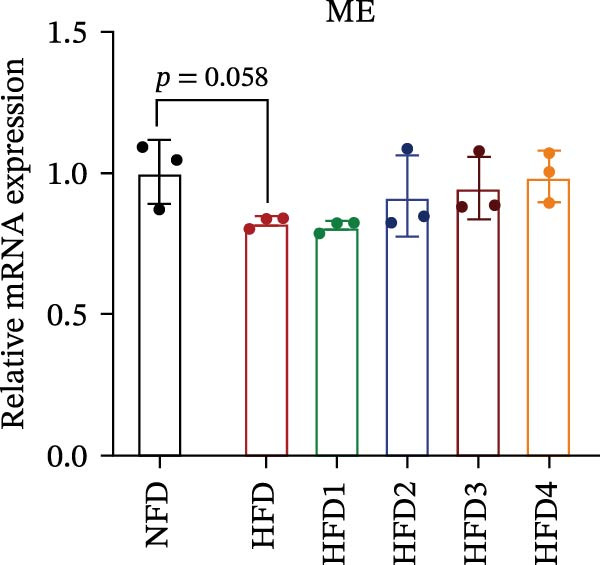
(E)

**Figure:**
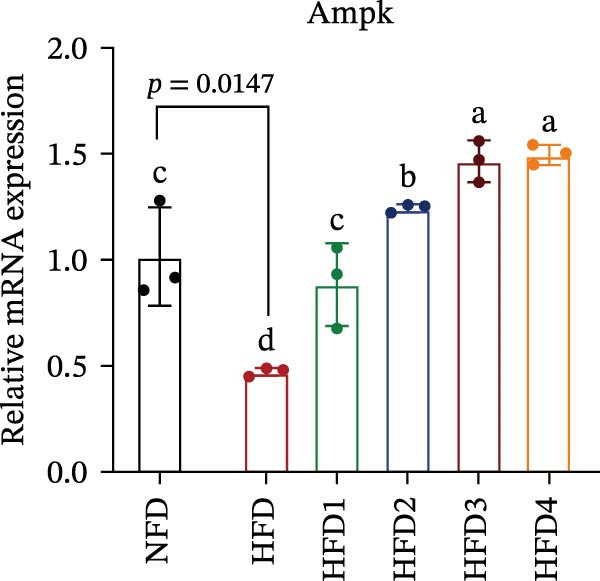
(F)

**Figure:**
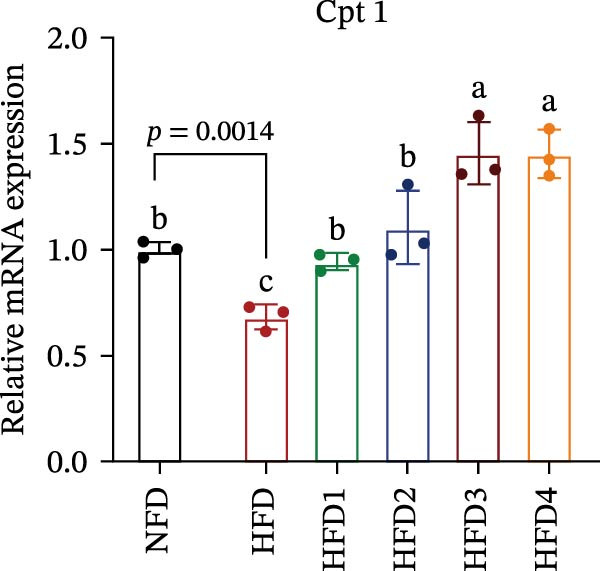
(G)

**Figure:**
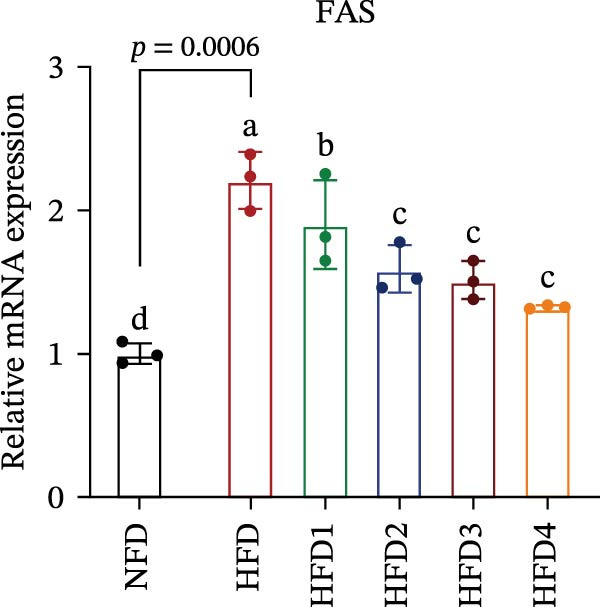
(H)

**Figure:**
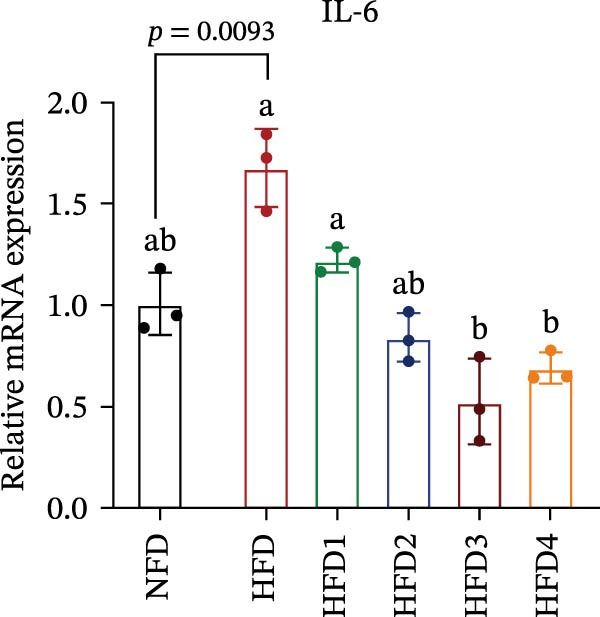
(I)

**Figure:**
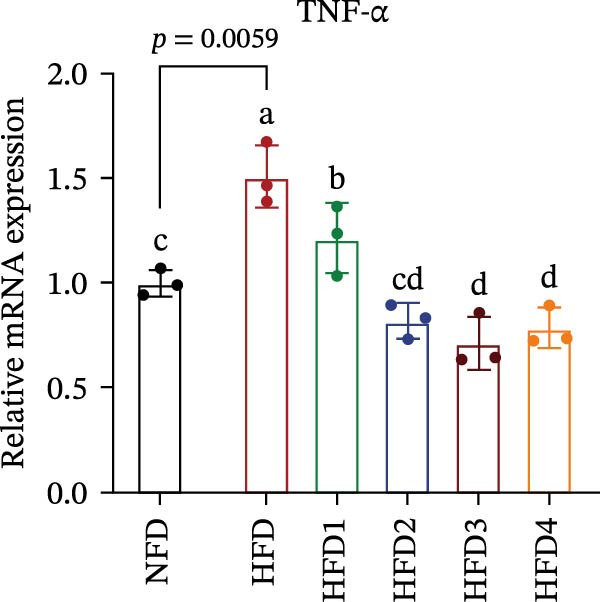
(J)

**Figure:**
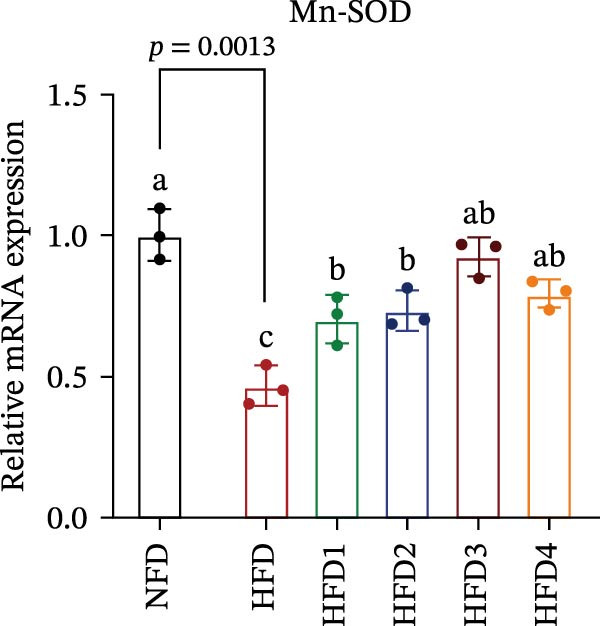
(K)

**Figure:**
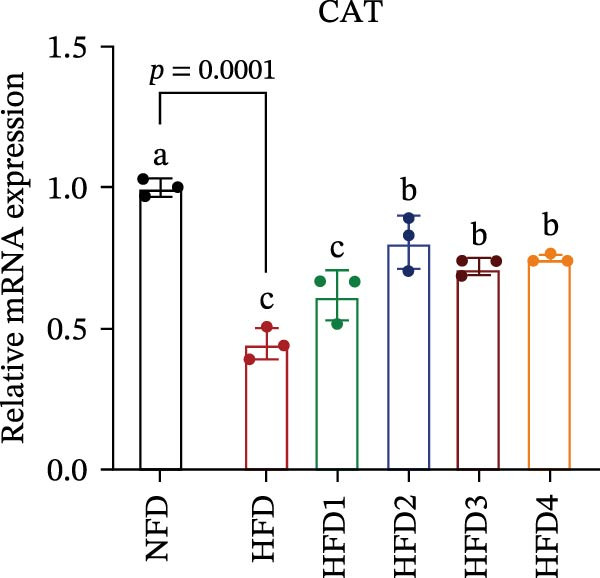
(L)

### 3.6. Intestinal Microbiome Analysis of *T. obscurus*

To investigate the effects of an HFD on the intestinal microbiota of *T. obscurus* and the changes induced by LBP supplementation, we conducted 16S rRNA sequencing on intestinal samples from six experimental groups, each with three replicates (18 samples in total). The rarefaction curve based on the coverage index (Figure 4D) revealed that the number of OTUs increased with sequencing depth and eventually plateaued. This finding confirms that the sequencing depth was sufficient to accurately capture the microbial diversity and composition of the intestinal bacterial community, thereby ensuring the reliability of subsequent analyses.

Figure 4Analysis of intestinal microbiota in *T. obscurus*. (A) Pielou_J index. (B) Simpson index. (C) Shannon index. (D) Rarefaction curve analysis. (E) Relative abundance at the phylum level. (F) Relative abundance at the class level.

**Figure:**
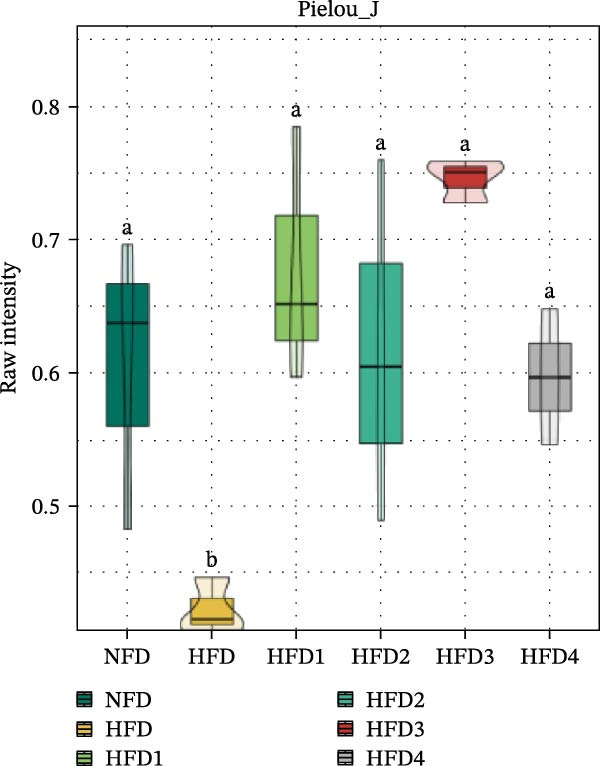
(A)

**Figure:**
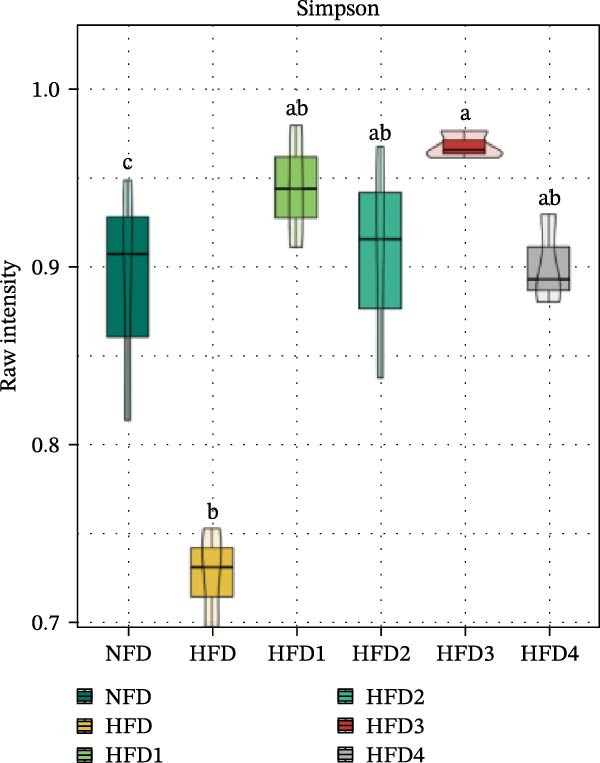
(B)

**Figure:**
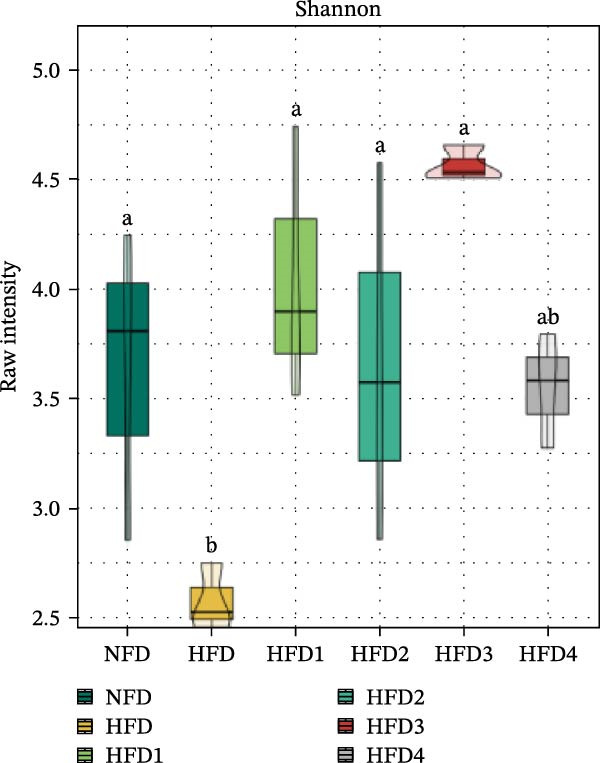
(C)

**Figure:**
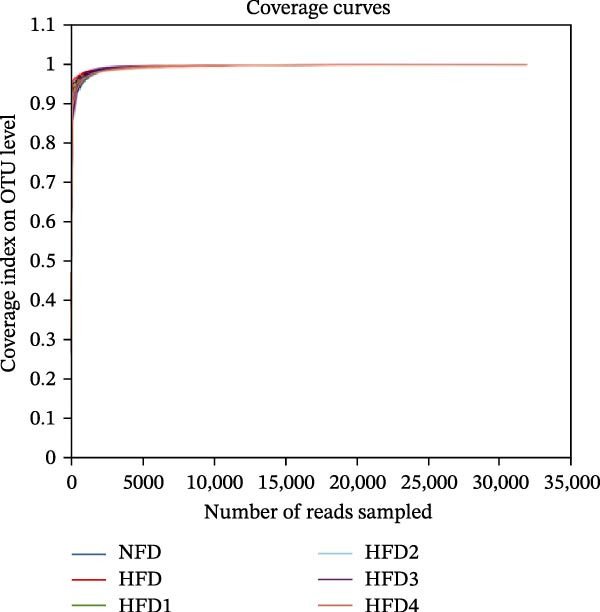
(D)

**Figure:**
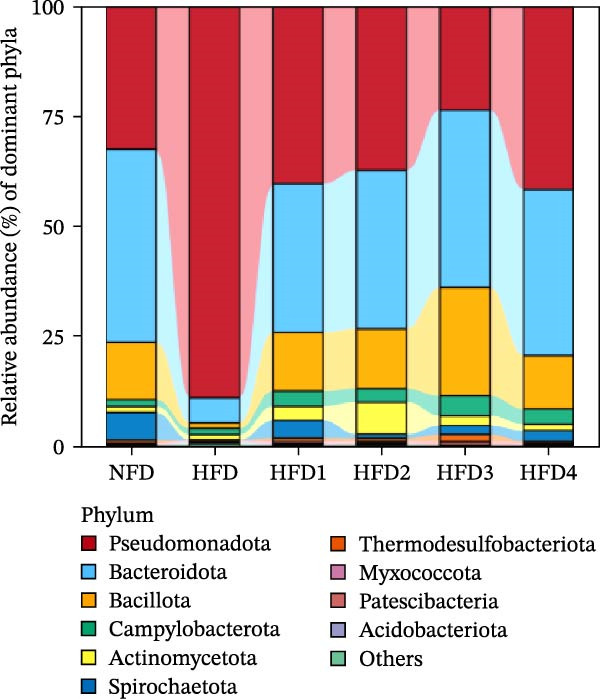
(E)

**Figure:**
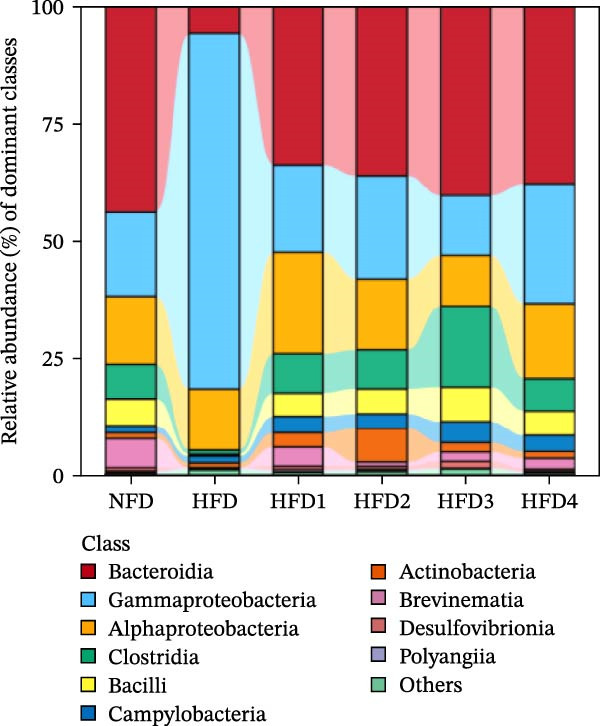
(F)

We then calculated the alpha diversity indices, including the Pielou, Simpson, and Shannon indices (Figure 4A–C), revealing that the Simpson index decreased after HFD feeding but increased with increasing levels of LBP supplementation, peaking in the HFD3 group (*p* < 0.05). In contrast, the Pielou and Shannon indices returned to normal levels following LBP supplementation.

To further assess the effects of LBP on gut microbiota composition under high‐fat conditions, we analyzed the dominant taxa at the phylum and class levels (top 10 in relative abundance). At the phylum level (Figure 4E), the dominant microbiota in the NFD group were primarily Bacteroidota and Pseudomonadota. In the HFD group, the relative abundance of Pseudomonadota notably increased, and this trend intensified with elevated dietary fat levels. However, LBP supplementation gradually restored microbial balance, as evidenced by the decrease in the abundance of Pseudomonadota and the concurrent increase in that of Bacillota. Notably, in the HFD3 group, the abundance of Pseudomonadota was the lowest, whereas that of Bacillota peaked, indicating that LBP effectively alleviated HFD‐induced gut microbial dysbiosis. At the class level (Figure 4F), Bacteroidia was dominant in the NFD group, whereas Bacteroidia, Gammaproteobacteria, and Alphaproteobacteria became predominant following HFD feeding. With increasing levels of LBP supplementation, the relative abundance of Bacteroidia increased, whereas those of Gammaproteobacteria and Alphaproteobacteria decreased. In addition, compared with the HFD group, the relative abundance of Clostridia was significantly elevated in the HFD3 group.

Collectively, these results indicate that the HFD3 group exhibited better overall performance than the other groups. Therefore, we constructed a differential microbiota heatmap (Supporting Information 5: Figure S4 and Supporting Information 6: Figure S5) using the NFD, HFD, and HFD3 groups; generated violin plots for key significant differential taxa; and conducted significance testing to assess differences. Supporting Information 5: Figure S4 and Supporting Information 6: Figure S5 detail all differential bacterial taxa identified at the phylum and class levels. As illustrated in Figure 5, following HFD feeding, the relative abundances of c_Bacilli, c_Bacteroidota, and p_Bacteroidia were significantly reduced (*p*  < 0.05), whereas those of Pseudomonadota and Gammaproteobacteria were significantly increased (*p*  < 0.05). However, following LBP supplementation, the HFD3 group exhibited significantly higher abundances of Bacteroidales, Clostridia, Bacillota, and Bacilli and significantly lower abundances of Gammaproteobacteria and Pseudomonadota than the HFD group (*p* < 0.05).

Figure 5Violin plot of key differential taxa of NFD vs. HFD vs. HFD3. (A–G) Violin plots showing the abundance of selected differential bacteria at the phylum and class levels among NFD, HFD, and HFD3 groups. Different letters indicate significant differences (*p*  < 0.05).

**Figure:**
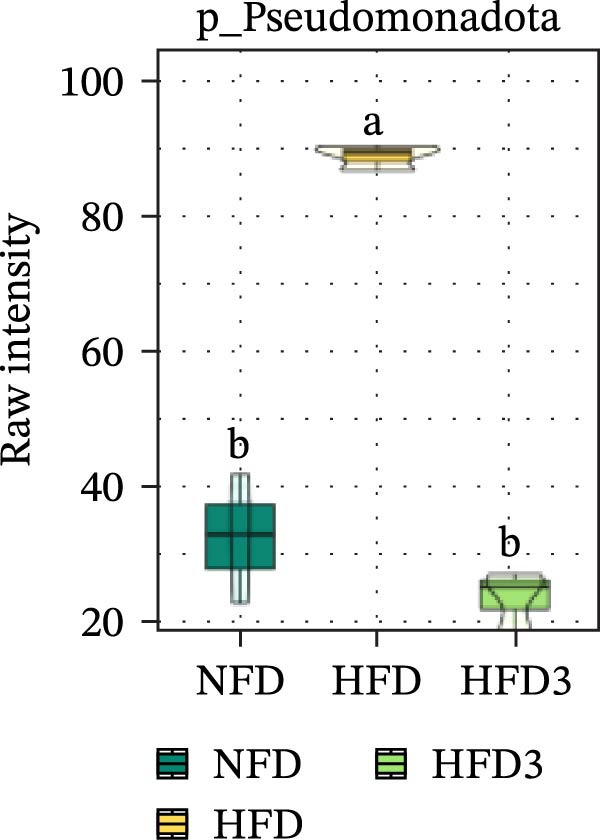
(A)

**Figure:**
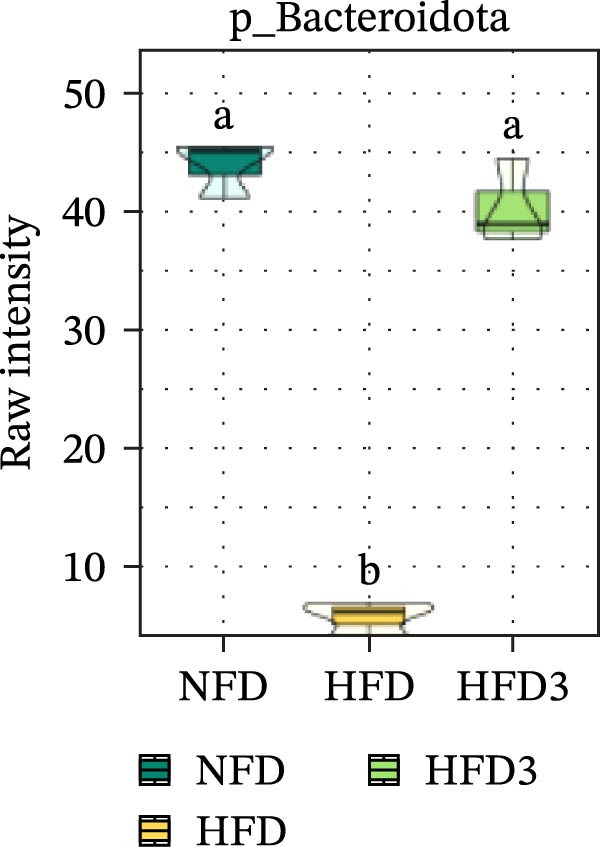
(B)

**Figure:**
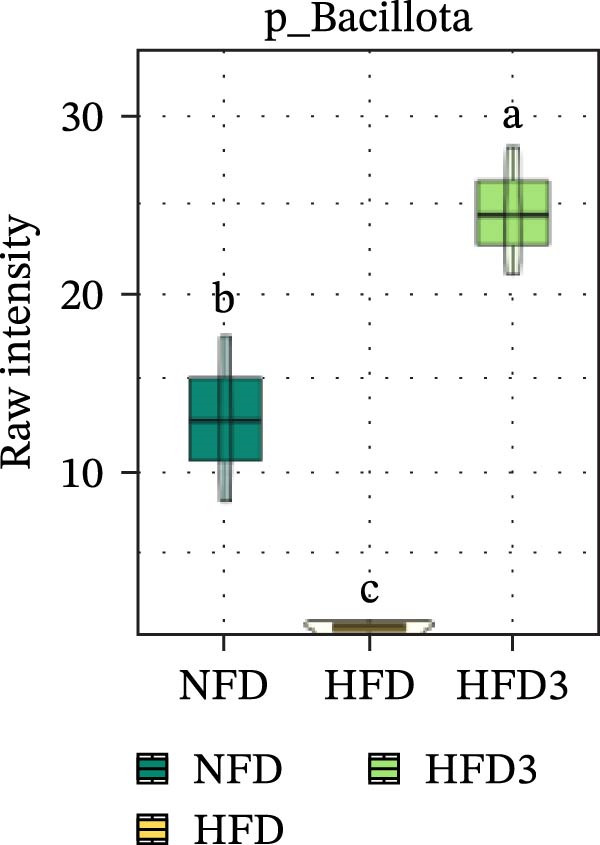
(C)

**Figure:**
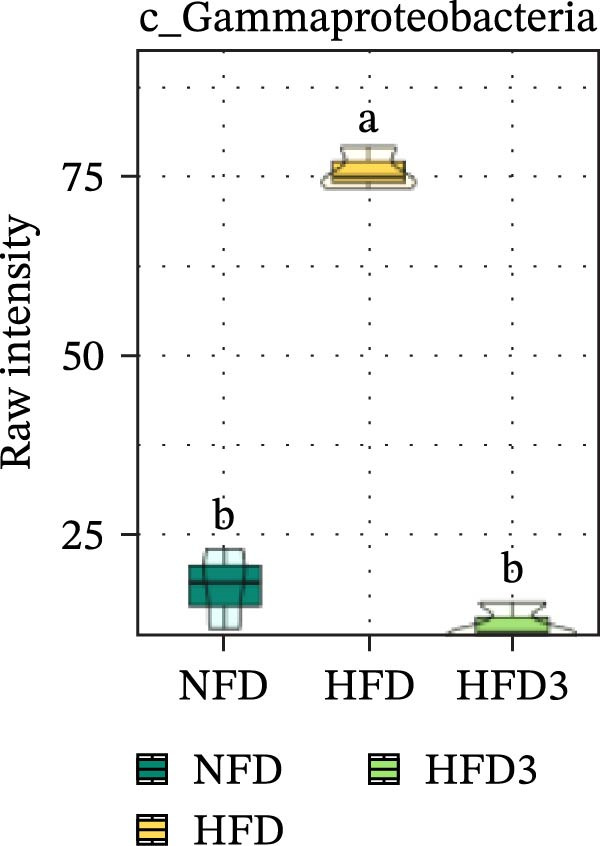
(D)

**Figure:**
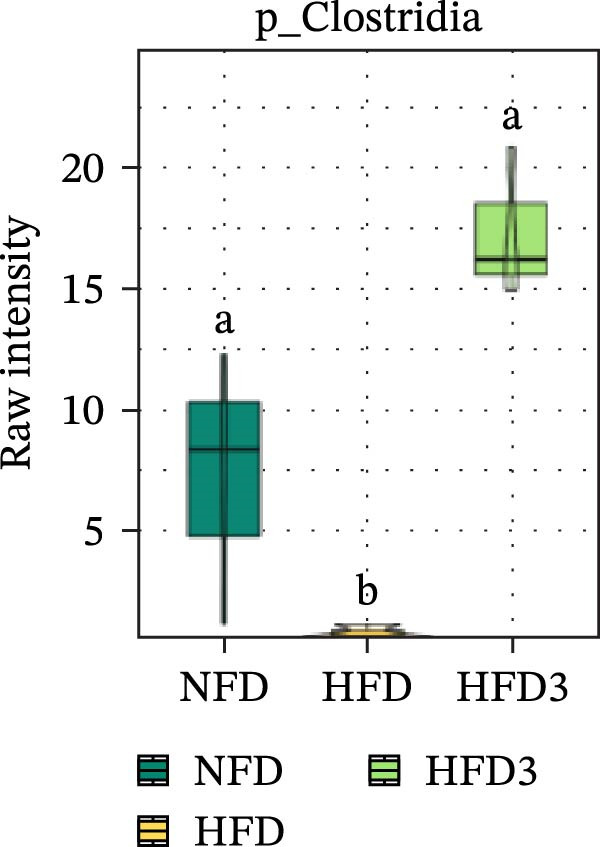
(E)

**Figure:**
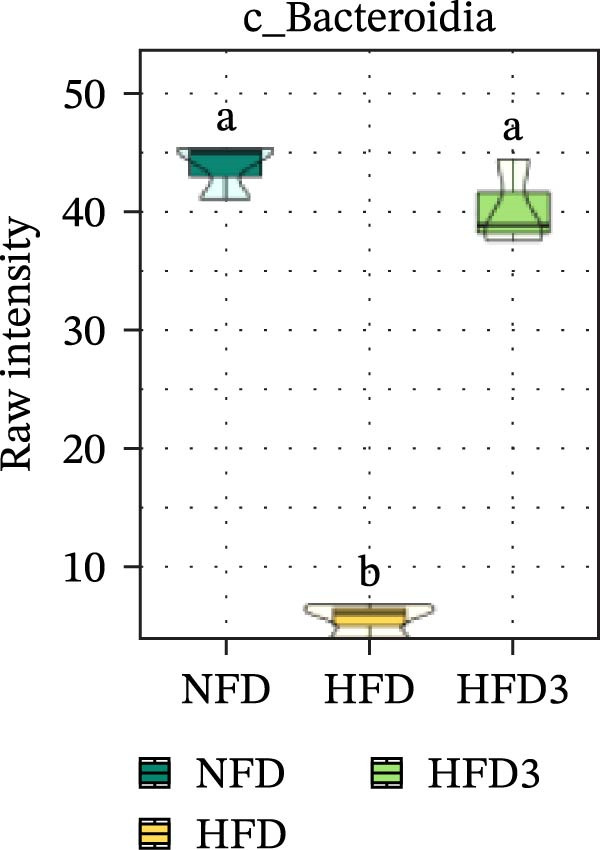
(F)

**Figure:**
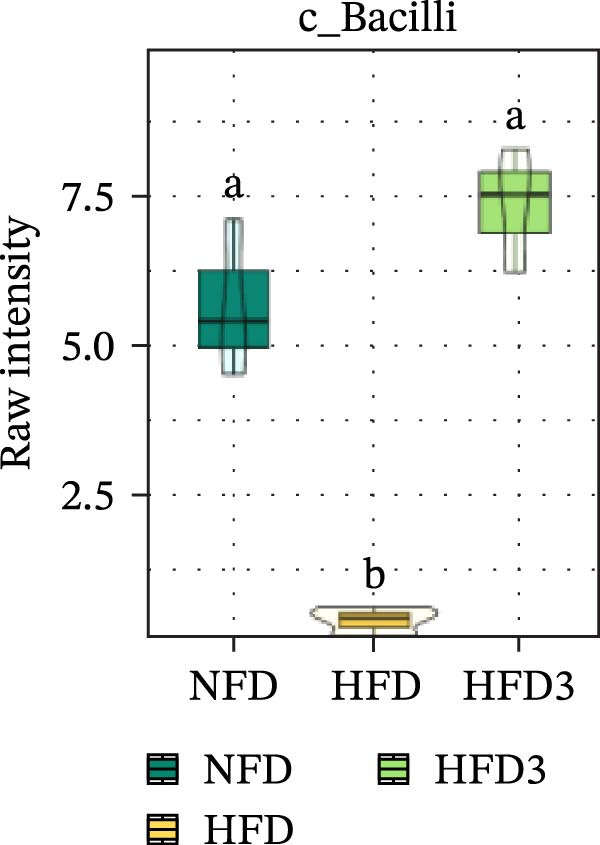
(G)

In summary, an HFD disrupted the gut microbiota of *T. obscurus*, whereas 1.5 g/kg LBP (HFD3) most effectively restored microbial diversity and promoted a healthier community structure.

### 3.7. Liver Metabolomics Analysis

To further investigate the effect of HFD on hepatic lipid metabolism in *T. obscurus* and the effect of LBP supplementation on hepatic metabolic profiles under high‐fat dietary conditions, we selected the NFD, HFD, and HFD3 groups for metabolomic analysis. Each group included six biological replicates, resulting in 18 liver samples.

#### 3.7.1. Principal Component Analysis (PCA) and Venn Diagram of Differential Hepatic Metabolites Among Groups

PCA score plots revealed clear metabolic separation among the NFD, HFD, and HFD3 groups (PC1: 26.12% and PC2: 18.98%; Figure 6A). Notably, the HFD3 group clustered closer to the NFD group along PC1 compared to the HFD group, suggesting that LBP supplementation partially restored the hepatic metabolic profile.

Figure 6(A) PCA scatter plot of hepatic metabolomic profiles in *T. obscurus*. (B) Venn diagram of differential hepatic metabolites in *T. obscurus*.

**Figure:**
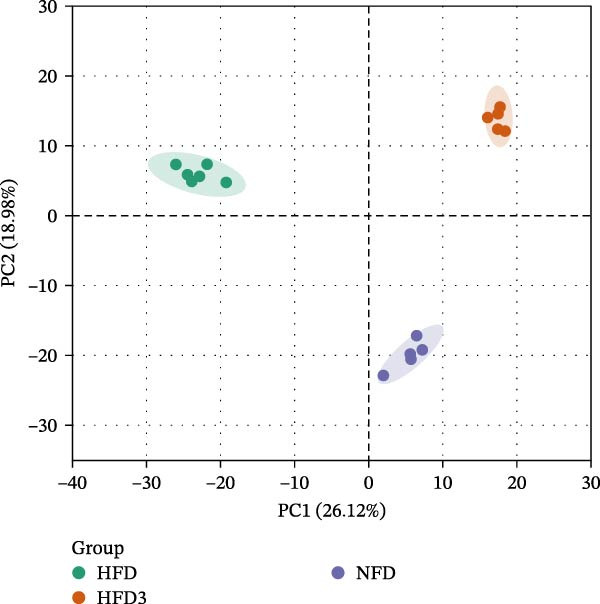
(A)

**Figure:**
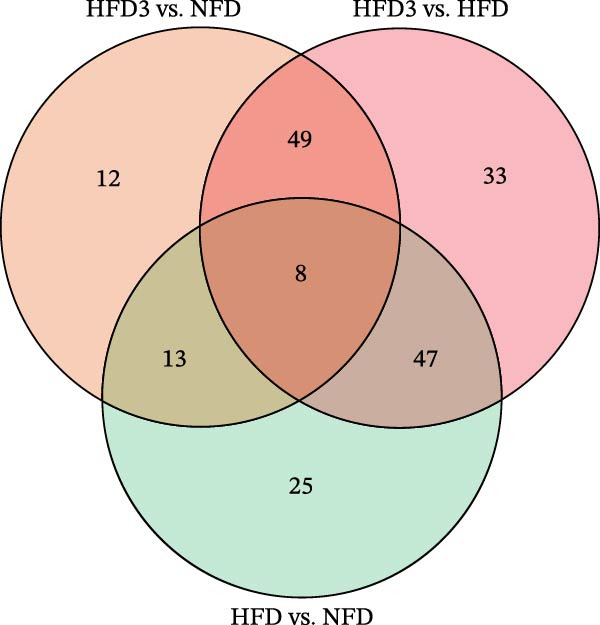
(B)

We also identified a total of 82 differential metabolites between the HFD3 and NFD groups, 137 between the HFD3 and HFD groups, and 93 between the HFD and NFD groups (Figure 6B). Notably, eight differential metabolites were shared among all three comparison groups, as indicated in the Venn diagram.

#### 3.7.2. Analysis of Differential Metabolites Across Comparison Groups in the Liver

In the OPLS‐DA, we observed clear separations among the liver samples of the HFD and NFD, HFD3 and HFD, and HFD3 and NFD groups. The OPLS‐DA models were validated using a 200‐iteration permutation test to ensure their robustness and prevent overfitting. As illustrated in Figure 7A–C, the *Q* ^2^ regression lines showed negative intercepts (all *Q* ^2^ intercepts < 0), and all permuted *R* ^2^ and *Q* ^2^ values were lower than the original values. These rigorous validation results indicate that the models are highly reliable for the identification of differential metabolites.

Figure 7Permutation tests of the OPLS‐DA model (A–C), volcano plots (D–F), and heatmaps (G–I) of differential hepatic metabolites in *T. obscurus*.

**Figure:**
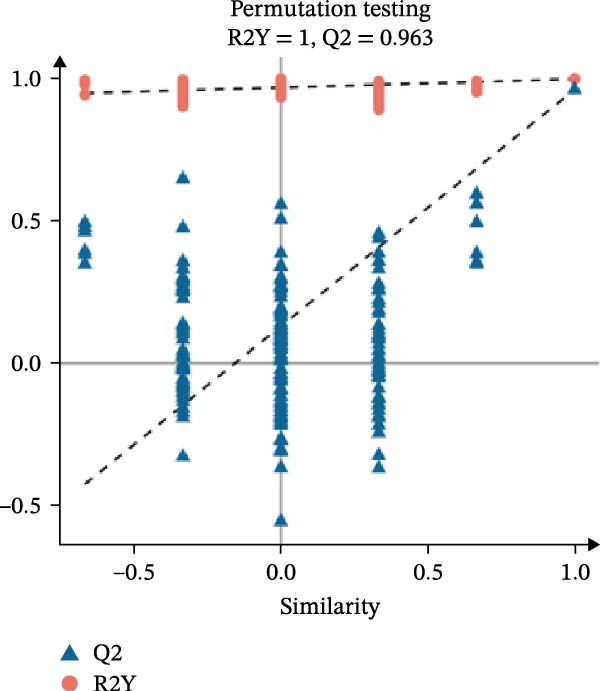
(A)

**Figure:**
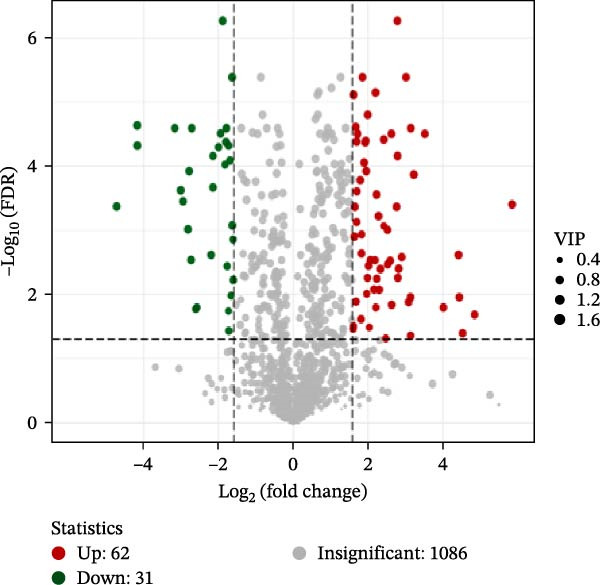
(B)

**Figure:**
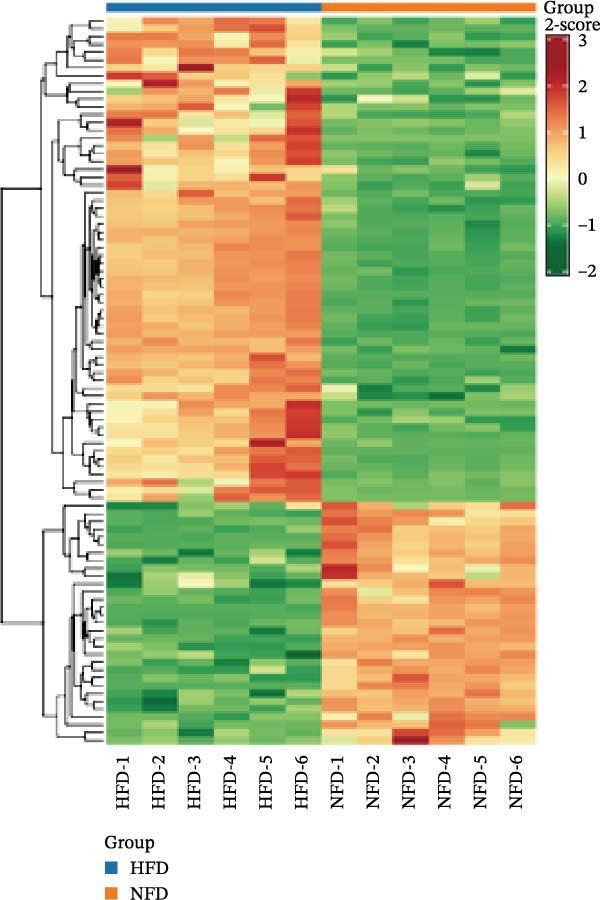
(C)

**Figure:**
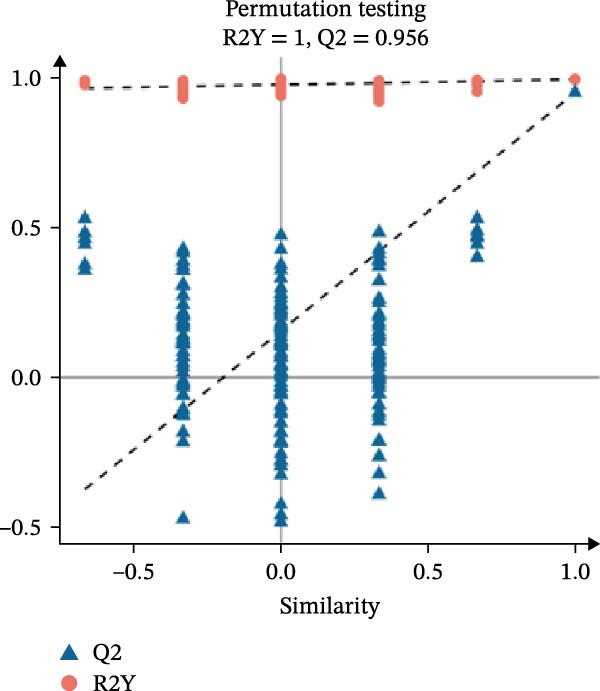
(D)

**Figure:**
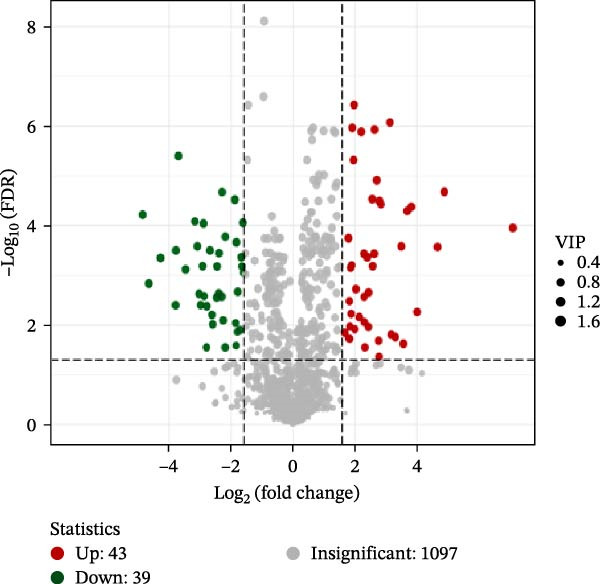
(E)

**Figure:**
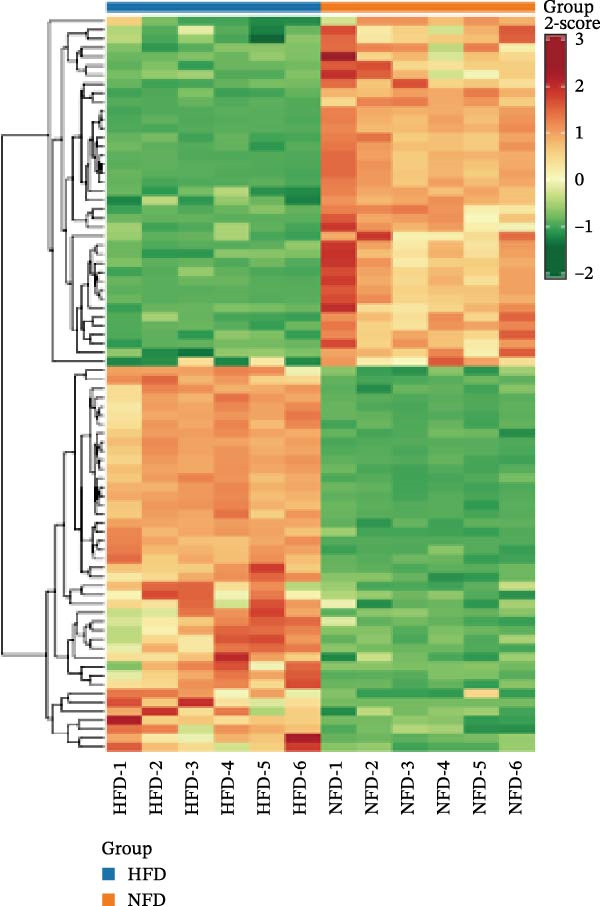
(F)

**Figure:**
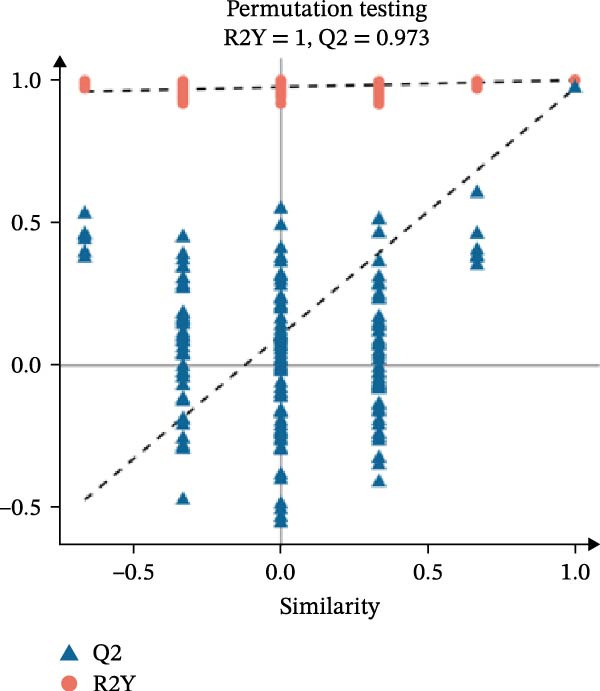
(G)

**Figure:**
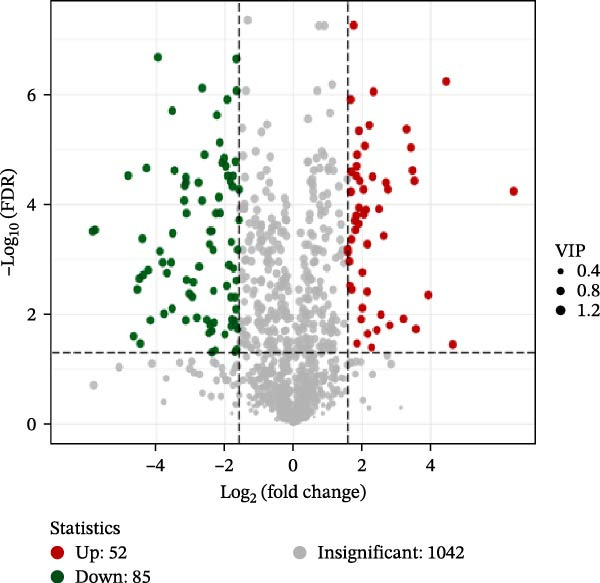
(H)

**Figure:**
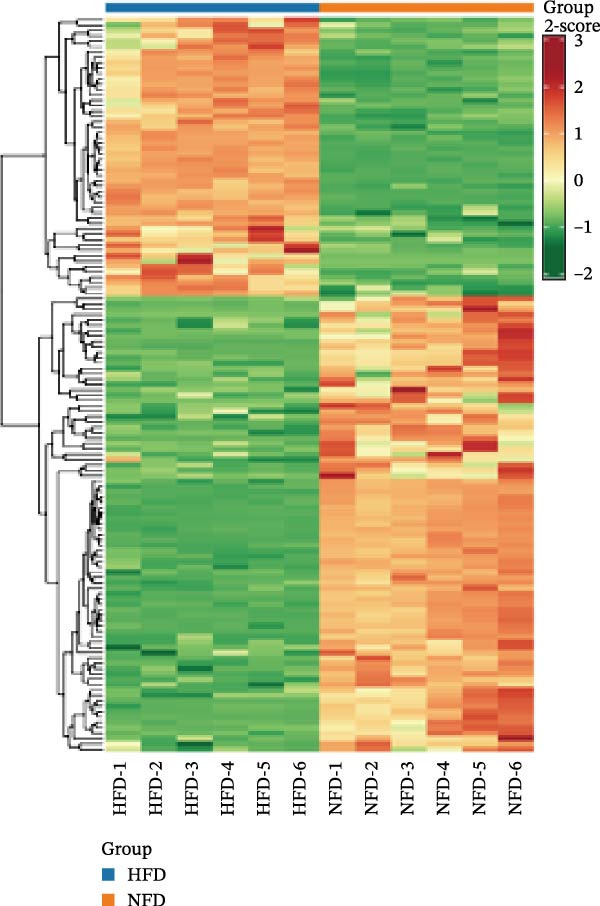
(I)

To identify differential metabolites between groups, we applied the following criteria of VIP > 1, *p*  < 0.05, and fold change ≥ 3 or ≤0.333. As a result, we identified 93 differential metabolites between the HFD and NFD groups, including 62 upregulated and 31 downregulated ones; 82 differential metabolites between the HFD3 and NFD groups, including 43 upregulated and 39 downregulated metabolites; and 137 differential metabolites between the HFD3 and HFD groups, including 52 upregulated and 82 downregulated metabolites (Figure 7D–F).

To visually illustrate the classification and expression differences of the metabolites, we generated heatmaps of the identified differential metabolites (Figure 7B,E,H; the magnitude of expression differences is represented by color intensity). The results indicated that differential metabolites between the HFD and NFD groups were mainly associated with organic acids and derivatives, nucleotides and their metabolites, glycerophospholipids, and bile acids (Figure 7G). Differential metabolites between the HFD3 and HFD groups were primarily enriched in glycerophospholipids, organic acids and derivatives, and heterocyclic compounds (Figure 7H). Differential metabolites between the HFD3 and NFD groups were predominantly enriched in glycerophospholipids, organic acids and derivatives, aldehydes, ketones, esters, and bile acids (Figure 7I). A detailed list of these differential metabolites, presented in the corresponding heatmaps, is provided in Supporting Information 7: Figure S6, Supporting Information 8: Figure S7, and Supporting Information 9: Figure S8.

##### 3.7.2.1. Bar Plot of Fold Changes in Differential Hepatic Metabolite

Using strict criteria (VIP > 1, *p*  < 0.05, and FC ≥ 3 or ≤0.333), 20 differential metabolites were identified (Figure 8). HFD significantly elevated bile acids (e.g., TCDCA and TDCA) and specific glycerophospholipids while downregulating organic acids and nucleotides compared to the NFD group. Notably, LBP supplementation reversed these trends by upregulating glycerophospholipids and downregulating bile acids and inflammatory markers. In the HFD3 group vs. NFD, residual differences were primarily characterized by upregulated lysophospholipids and downregulated benzenoids and organic acids (e.g., angiotensin I/II).

Figure 8Bar plots of fold changes in differential hepatic metabolites of *T. obscurus*. (A) HFD vs. NFD. (B) HFD3 vs. NFD. (C) HFD3 vs. HFD.

**Figure:**
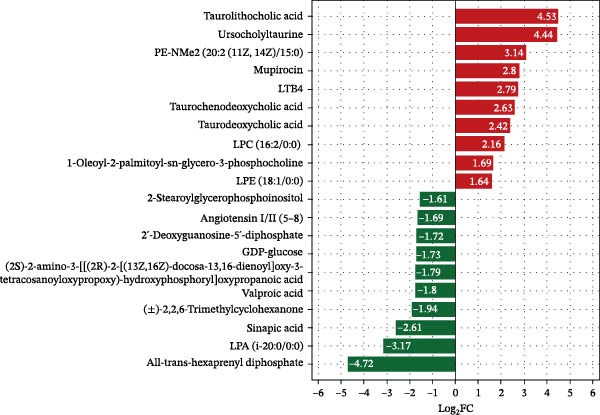
(A)

**Figure:**
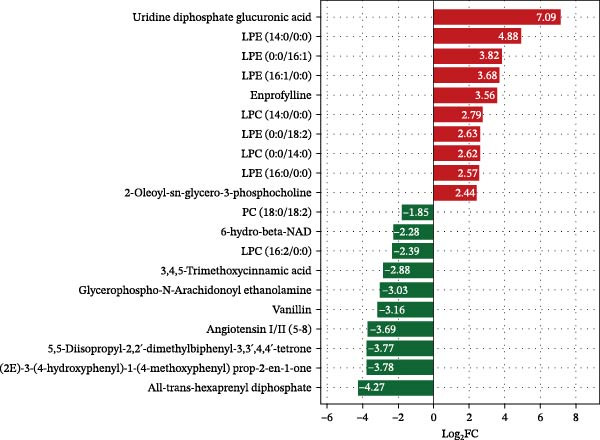
(B)

**Figure:**
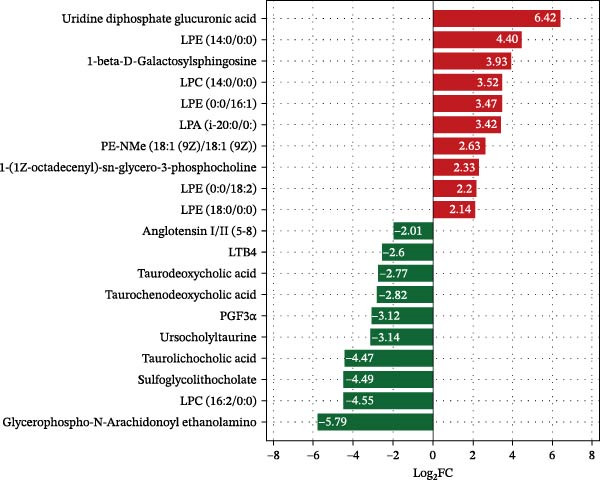
(C)

We then visualized bile acid‐related differential metabolites that were commonly altered across the HFD, NFD, and HFD3 groups (based on VIP > 1, *p*  < 0.05, and FC ≥ 3 or ≤0.333) using violin plots and analyzed them for statistical significance. As depicted in Figure 9, following LBP supplementation, all bile acid metabolites returned to normal levels (*p* < 0.05), except for glycocholic acid, which exhibited no significant difference from its level in the HFD group (*p* > 0.05).

Figure 9Violin plots of differential bile acid metabolites among NFD, HFD, and HFD3 groups. (A–J) Violin plots displaying the levels of all identified differential bile acids across the NFD, HFD, and HFD3 groups. Different letters indicate significant differences (*p*  < 0.05).

**Figure:**
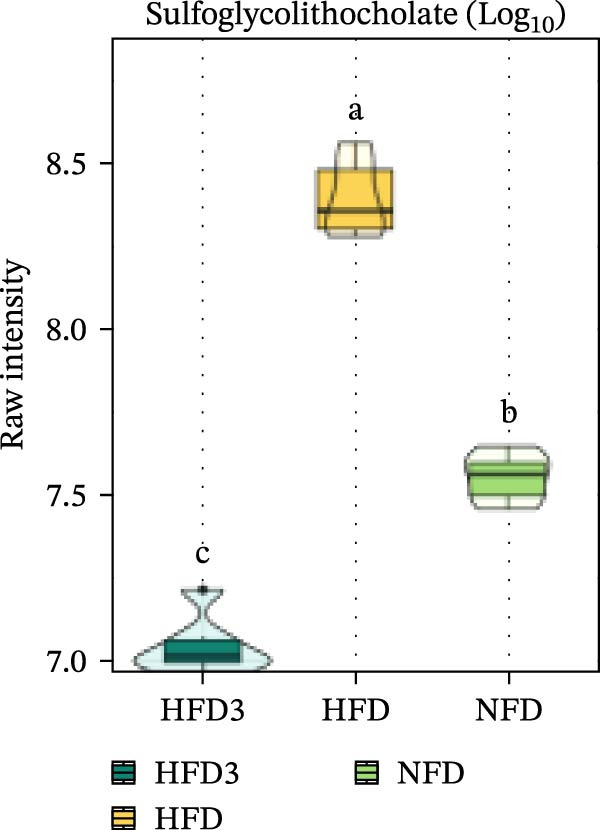
(A)

**Figure:**
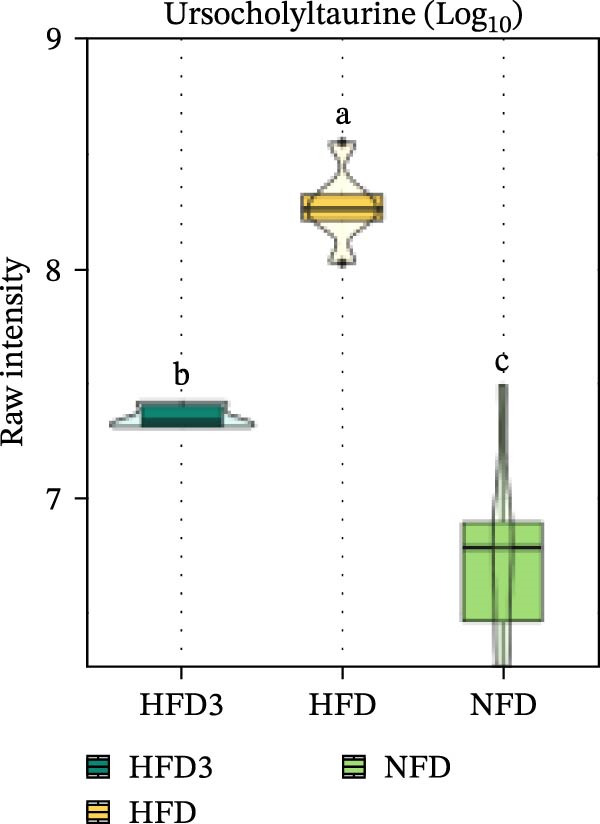
(B)

**Figure:**
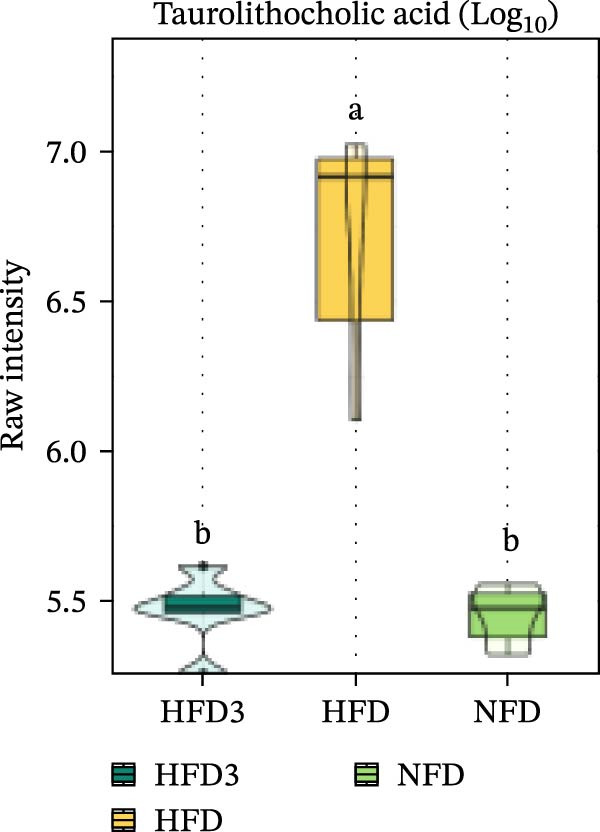
(C)

**Figure:**
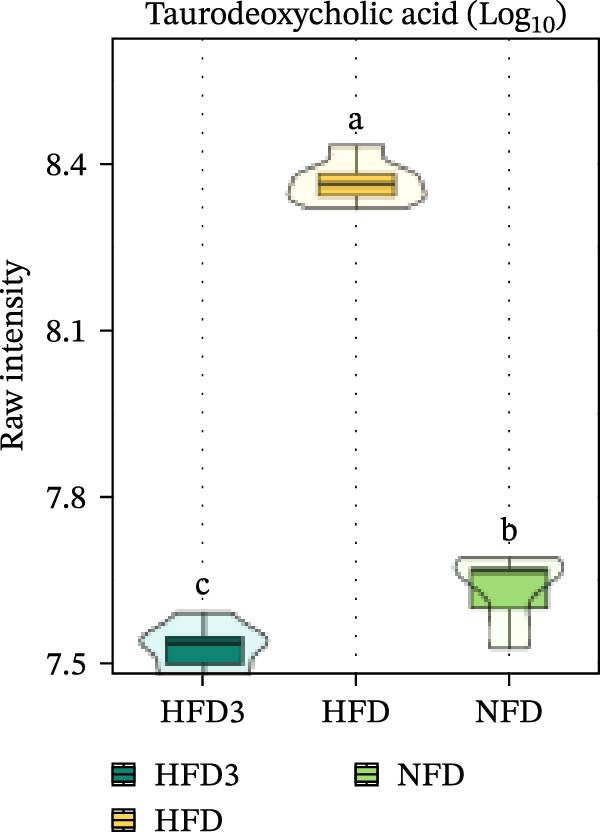
(D)

**Figure:**
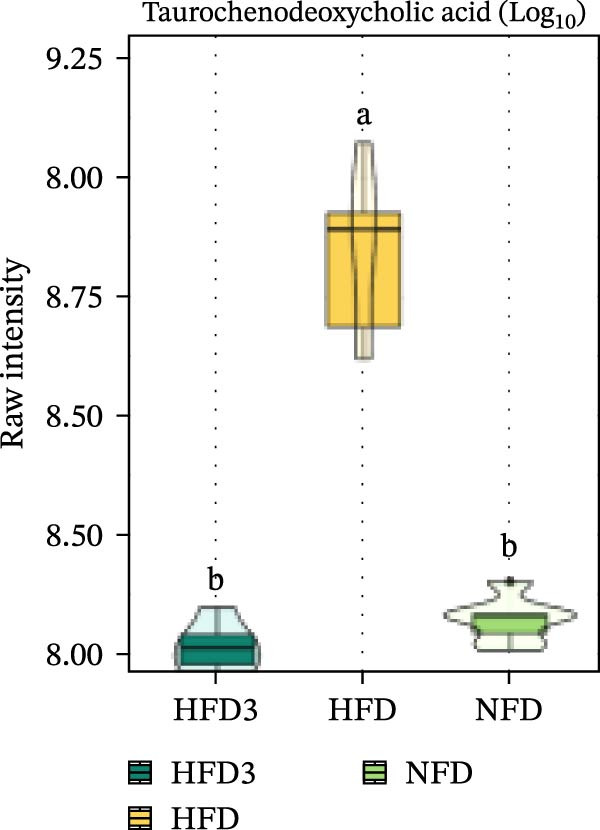
(E)

**Figure:**
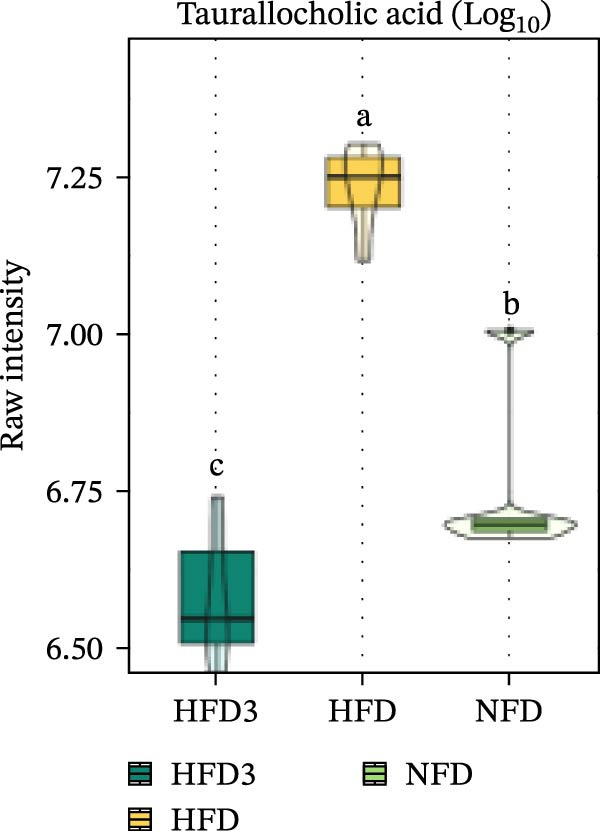
(F)

**Figure:**
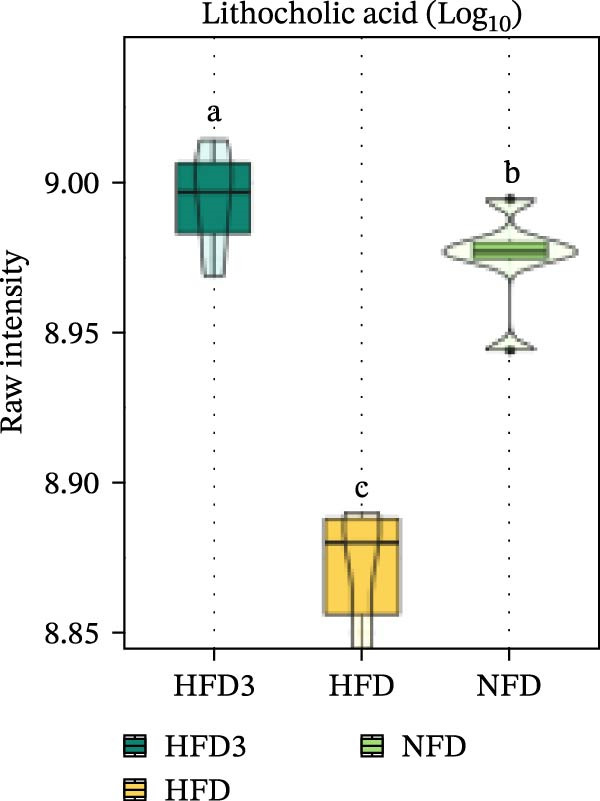
(G)

**Figure:**
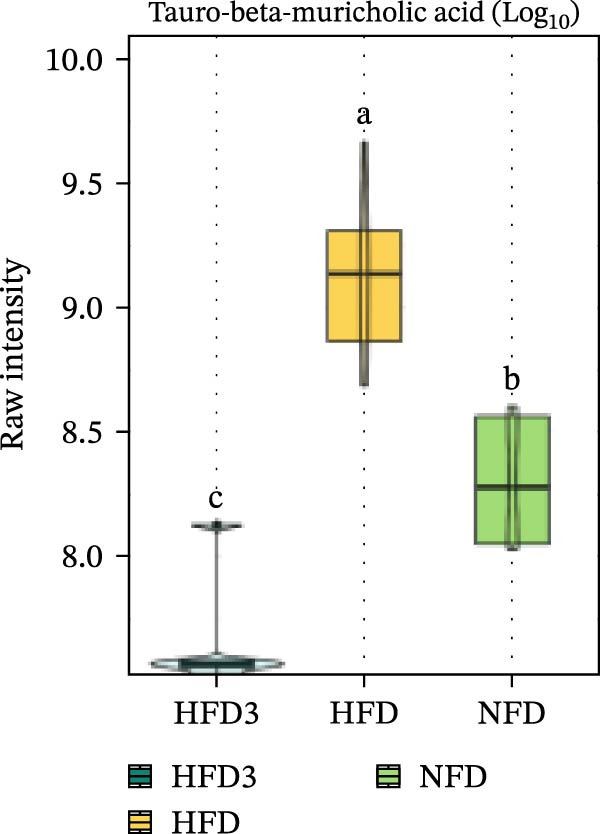
(H)

**Figure:**
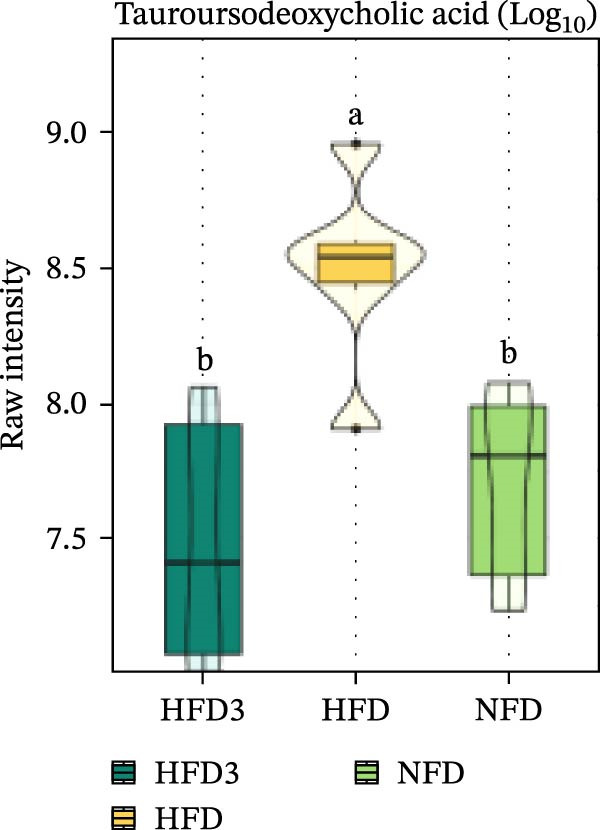
(I)

**Figure:**
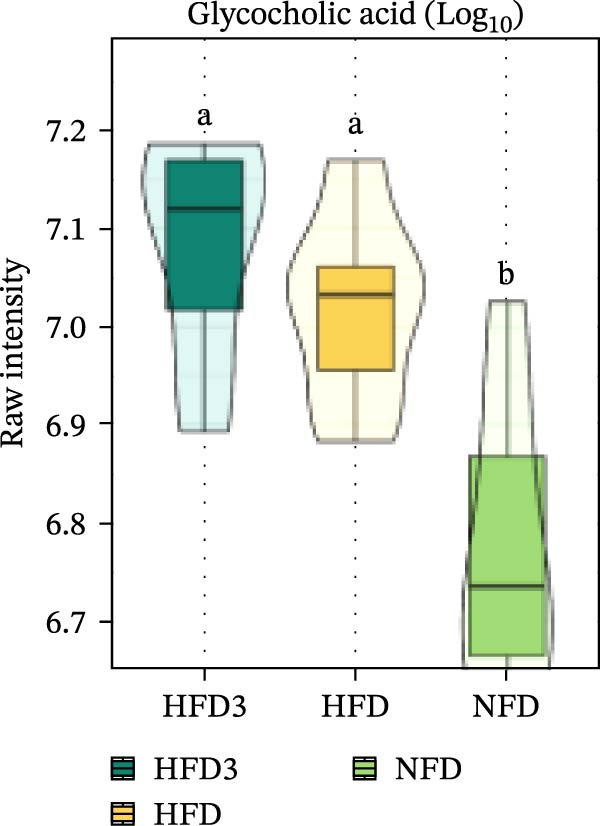
(J)

KEGG enrichment analysis identified key metabolic pathways among groups (Figure 10). HFD primarily disrupted propanoate metabolism, bile secretion, pyruvate metabolism, and the renin–angiotensin system. Notably, LBP supplementation (HFD3 vs. HFD) significantly modulated lipid‐related pathways, including arachidonic acid, glycerophospholipid, and linoleic/*α*‐linolenic acid metabolism. Interestingly, the HFD3 vs. NFD comparison revealed enrichment in the same four pathways as the HFD vs. NFD group, suggesting that LBP primarily targets these core HFD‐induced metabolic disruptions.

Figure 10Enriched metabolic pathways of hepatic metabolites in *T. obscurus*. (A) HFD vs. NFD. (B) HFD3 vs. NFD. (C) HFD3 vs. HFD.

**Figure:**
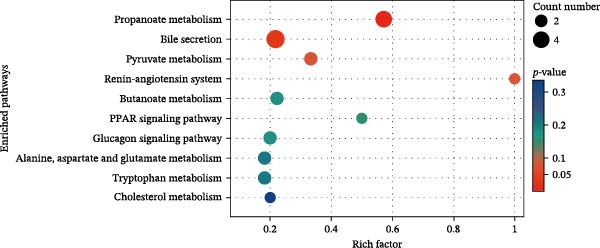
(A)

**Figure:**
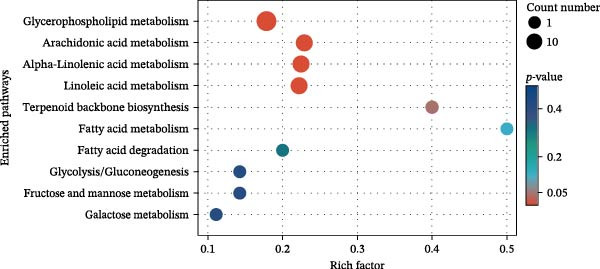
(B)

**Figure:**
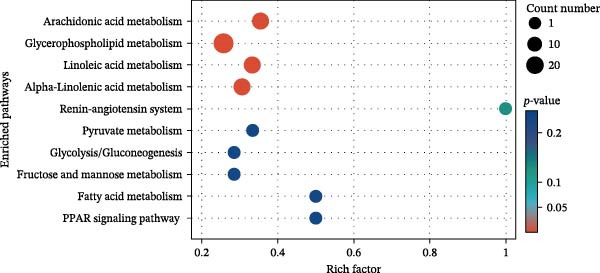
(C)

### 3.8. Correlation Among Gut Microbiota, Hepatic Metabolites, and Gene Expression

Spearman correlation analysis was performed to explore the link between gut microbiota and hepatic metabolites (Figure 11A). Specifically, p_Pseudomonadota showed significant positive correlations (*p*  < 0.05) with inflammatory markers (e.g., LTB4 and angiotensin I/II) and bile acids (e.g., tauro‐*β*‐muricholic and taurolithocholic acids). Conversely, it was negatively correlated with lysophosphatidic acid (i‐20:0/0:0). These results highlight a strong association between LBP‐modulated microbiota and the hepatic metabolic profile. Moreover, c_Gammaproteobacteria was significantly positively correlated (*p*  < 0.05) with LTB4, angiotensin I/II (5–8), and glycerophospho‐N‐arachidonoyl ethanolamine but negatively correlated with glycerophospholipids, including lysophosphatidylethanolamine (18:0/0:0) and lysophosphatidylethanolamine (14:0/0:0). c_Bacilli was significantly negatively correlated (*p*  < 0.05) with bile acid metabolites, such as taurochenodeoxycholic acid, taurolithocholic acid, taurodeoxycholic acid, and prostaglandin F3*α*. In addition, p_Bacteroidales was significantly negatively correlated (*p*  < 0.05) with angiotensin I/II (5–8), LTB4, and taurochenodeoxycholic acid. Similarly, c_Bacteroidia and p_Bacillota exhibited significant negative correlations (*p*  < 0.05) with bile acid metabolites, including sulfoglycolithocholate, taurochenodeoxycholic acid, taurallocholic acid, and ursocholyltaurine. Collectively, these results suggest that LBP may alleviate lipid metabolism disorders and bile acid dysregulation in HFD‐fed *T. obscurus* by modulating the abundance of key microbial taxa, such as p_Bacteroidales, p_Bacillota, p_Pseudomonadota, c_Gammaproteobacteria, c_Bacilli, and c_Clostridia.

Figure 11Integrative analysis of gut microbiota, hepatic metabolomics, and gene expression. (A) Cluster heatmap of differential metabolites and microbial taxa. (B) Mantel test analysis of differential metabolites, microbial taxa, and gene expression.

**Figure:**
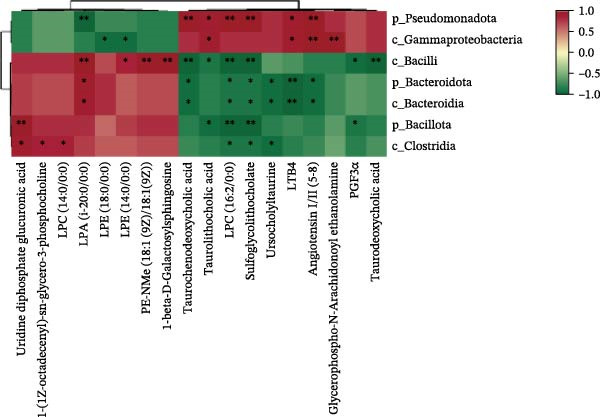
(A)

**Figure:**
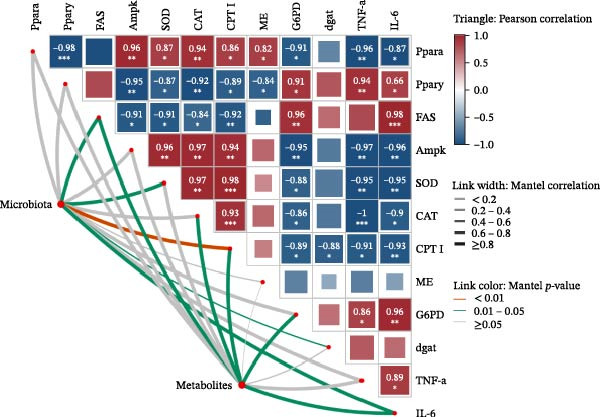
(B)

Finally, we performed a Mantel test analysis to assess potential correlations among differential metabolites, microbial taxa, and gene expression profiles between the HFD and HFD3 groups. As illustrated in Figure 11B, the gut microbiota was strongly correlated (*p* < 0.05) with the mRNA expression of *Fas*, *Mn-Sod*, *Cpt1*, *Dgat*, and *IL-6*, while metabolites were correlated (*p* < 0.05) with *Fas*, *Cat*, *Cpt1*, *G6pd*, and *IL-6*.

## 4. Discussion

Previous studies have shown that excessive dietary fat intake in fish can cause a range of adverse effects, including reduced growth, suppressed appetite, liver damage, immunosuppression, and excessive lipid accumulation [39, 40]. Based on these findings, in the present study, we used a diet containing ~150 g/kg lipids to induce lipid metabolism disorders in *T. obscurus*. Our results indicated that HFD‐fed *T. obscurus* individuals exhibited reduced growth rate, elevated serum lipid levels, hepatic injury, and severe hepatic lipid accumulation. These results indicate that although *T. obscurus* requires a lipid‐rich diet, an excessive HFD does not improve its growth but instead inhibits it. Notably, we demonstrated that supplementing the HFD with an appropriate amount of LBP partially improved the growth rate of *T. obscurus*, consistent with the findings of Tan et al. [25] and Huang et al. [30]. Elevated HSI and VSI are linked to reduced growth in fish, suggesting hepatic dysfunction or excessive hepatic lipid accumulation [41]. In the present study, consumption of an HFD significantly increased both the HSI and VSI in *T. obscurus*, whereas dietary supplementation with LBP reduced both indices. Notably, LBP can enhance lipid utilization efficiency by downregulating the expression of lipid synthesis‐related genes, including *Fas*, *Srebp-1*, and *Ppar-γ* [42]. Moreover, the improvements in lipid metabolism were accompanied by shifts in the gut microbiota, specifically the proliferation of Bacteroidetes and increased short‐chain fatty acid (SCFA) production, which were closely correlated with the observed reductions in HSI and VSI [43, 44]. Appropriate LBP supplementation partially restores growth and alleviates HFD‐induced hepatic lipid accumulation and metabolic disorders in *T. obscurus*.

The bioactivity of natural plant polysaccharides is closely related to their molecular weight and structure. For example, polysaccharides with molecular weights ranging between 10 and 100 kDa exhibit favorable bioactivity in regulating the gut microbiota and blood glucose levels [45]. Moreover, glucose and galactose can improve intestinal mucosal integrity, maintain the intestinal barrier, increase the Firmicutes/Bacteroidetes ratio, and promote SCFA production, thereby supporting intestinal health and physiological function [45]; notably, this may represent one mechanism through which LBP exerts its beneficial effects. However, owing to the structural complexity of polysaccharides, with their bioactivity influenced by the composition of monosaccharides, glycosidic linkage patterns, branching degrees, and molecular weight, further research is warranted to elucidate the precise mechanisms involved.

In the present study, HFD feeding significantly increased serum TG, TBA, TCHO, AST, ALT, and liver MDA levels while decreasing HDL‐C and T‐SOD levels. Notably, LBP supplementation significantly reversed these effects. Consequently, elevated LDL or abnormally low HDL impairs hepatic lipid regulation and exacerbates oxidative stress [46]. Similarly, Tan et al. [25] reported that LBP supplementation significantly reduces serum TCHO, TG, and LDL in *E. lanceolatus♂ × E. fuscoguttatus♀*. Notably, our findings align with these results, as dietary LBP significantly reduced serum TCHO, TG, and LDL levels in *T. obscurus*. In addition, natural plant polysaccharides promote lipid metabolism through multiple mechanisms, including inhibition of exogenous lipid absorption. These polysaccharides can bind to lipid molecules or bile salts in the gastrointestinal tract, promoting TG metabolism and utilization [47]. In fish, high‐fat feeding induces hepatic cholesterol accumulation [48]. The liver converts excess cholesterol into bile acids, which are transported into the bloodstream via bile acid transporters, leading to elevated serum bile acid levels. AST and ALT are normally stored in hepatocytes; however, following cellular injury, they leak into circulation, making them key indicators of liver damage [49]. Notably, in the present study, LBP supplementation significantly reversed HFD‐induced increases in serum AST and ALT levels in *T. obscurus*. Elevated serum AST and ALT levels result from their release during hepatocyte damage, and their reduction following LBP supplementation indirectly indicates alleviated hepatic injury.

An HFD also increases the burden on the intestinal system, causing undigested lipids to accumulate in the liver [50], potentially leading to hepatic steatosis, inflammation, and fibrosis [42]. An HFD impairs mitochondrial respiratory chain complexes activity, resulting in excessive ROS production. Accumulated ROS suppresses antioxidant enzyme activity and reacts with lipids to generate MDA, thereby inducing oxidative stress [51]. In this study, HFD‐fed *T. obscurus* exhibited reduced hepatic T‐SOD activity and elevated MDA levels. However, LBP supplementation restored T‐SOD activity and normalized MDA concentrations. These findings are consistent with the results of Huang et al. [30], who reported that LBP improves antioxidant capacity and reduces MDA levels in HFD‐fed *L. maculatus*. These results further support the ability of polysaccharides, which possess hydroxyl and other reactive groups, to scavenge free radicals and inhibit hydroxyl radical‐induced lipid peroxidation [52].

As the central organ of lipid storage, metabolism, and detoxification [53], the liver is highly sensitive to dietary fat intake. An HFD may cause excessive visceral fat accumulation, leading to the release of various cytokines, such as TNF‐*α*, IL‐6, and IL‐1*β*, from adipose tissue, thus triggering hepatic inflammation [54]. Our results indicated that an HFD significantly altered hepatic morphology and caused prominent lipid vacuolization in *T. obscurus*. *Ppar-γ* regulates adipocyte differentiation and lipid synthesis, whereas FAS is a rate‐limiting enzyme in fatty acid biosynthesis. LBP can modulate lipid metabolism‐related gene transcription and suppresses *Ppar-γ* and *Fas* expression, thus alleviating lipid metabolism disorders [55]. It also may reduce de novo lipogenesis by inhibiting free fatty acid (FFA) uptake [56]. In our study, LBP supplementation significantly downregulated *Ppar-γ*, *G6pd*, and *Fas* expression but did not affect *Me* expression, likely because G6pd provides sufficient NADPH for fatty acid synthesis, maintaining ME stability [24]. Moreover, the HFD‐induced downregulated expression of *AMPK*, *Cpt1*, and *Ppar-α*, which are key transcriptional regulators of lipid metabolism that promote fatty acid oxidation [35], indicates disrupted hepatic lipid metabolism in *T. obscurus*. Notably, LBP supplementation restored the expression of these genes. Collectively, these results demonstrate that LBP promotes lipid metabolism and alleviates hepatic inflammation in *T. obscurus*.

Chronic HFD consumption compromises intestinal tight junction integrity, increases barrier permeability, and alters gut microbial composition [57]. Consistently, our results indicated that an HFD significantly disrupted the intestinal microbial structure of *T. obscurus*, increasing the abundance of harmful bacteria and reducing that of beneficial SCFA–producing taxa. Notably, LBP supplementation modulated the gut microbiota by suppressing pathogenic taxa (Pseudomonadota, Gammaproteobacteria, and Lysobacterales) and promoting beneficial ones (Bacillota, Bacteroidales, Lactobacillales, Clostridia, and Lachnospirales). These beneficial groups produce SCFAs [58], such as acetate and butyrate, which activate the AMPK pathway and inhibit SREBP signaling, thereby suppressing lipid synthesis [59]. This result is consistent with a previous study indicating that LBP can activate the AMPK signaling pathway and upregulate Cpt1 expression, thus enhancing fatty acid oxidation [60]. Furthermore, Clostridia contribute to the conversion of conjugated bile acids (CBAs) into secondary bile acids (SBAs), playing a critical role in enterohepatic circulation [61]. An HFD inhibits the farnesoid X receptor signaling pathway, leading to activation of cholesterol 7*α*‐hydroxylase (CYP7A1), the rate‐limiting enzyme in bile acid biosynthesis, thereby disrupting bile acid homeostasis and promoting hepatic fat accumulation [62]. Overall, these results indicate that LBP supplementation restored gut microbial balance, enhanced diversity, and normalized bile acid and glycerophospholipid metabolism.

In this study, excessive dietary fat substantially increased the levels of bile acids, leukotrienes, and related metabolites in *T. obscurus*. Consistent with these findings, KEGG enrichment analysis indicated that a HFD significantly disrupted multiple metabolic pathways. Notably, the simultaneous enrichment of propanoate and pyruvate metabolism, together with alterations in bile secretion, suggests that fish experienced pronounced metabolic stress under high lipid intake. Excess dietary fat may promote the accumulation of propionyl‐CoA, which has been shown to overload propionate metabolism and increase substrate flux into the tricarboxylic acid cycle (TCA), thereby exacerbating hepatic metabolic burden [63]. In the high‐fat group, enrichment of the bile secretion pathway not only reflects an increased demand for bile acid excretion in response to lipid overload but also suggests a possible disruption in bile acid synthesis and transport. This imbalance may contribute to abnormal bile acid metabolism [64]. Leukotrienes, produced during fatty acid peroxidation, are inflammatory mediators involved in hepatic inflammation [65]. Propionate and pyruvate are precursors of lipid synthesis and can be metabolized into acetyl‐CoA, which is then used for fatty acid and cholesterol biosynthesis [66]. CYP7A1 converts cholesterol into bile acids [67]; thus, an HFD increases substrate availability, enhancing bile acid production. Phospholipase A2 catalyzes the hydrolysis of membrane phospholipids, primarily phosphatidylcholine and phosphatidylethanolamine, to produce lysophospholipids, such as lysophosphatidylcholine and lysophosphatidylethanolamine, both of which are critical in lipid metabolism [68]. Uridine diphosphate glucuronic acid is an essential substrate in UDP‐glucuronosyltransferase reactions. Its upregulation influences TG metabolism by participating in glycolipid metabolic pathways. In addition, UDP‐glucuronosyltransferases are involved in TBA metabolism, thereby indirectly affecting lipid regulation [69]. Moreover, the *α*‐linolenic acid metabolic pathway plays a crucial role in modulating inflammation and maintaining fatty acid homeostasis. It primarily suppresses the SREBP family, reducing the expression of genes involved in cholesterol and TG synthesis while simultaneously activating the AMPK signaling pathway and upregulating genes related to fatty acid oxidation. Collectively, these changes promote lipid metabolism [70]. Furthermore, the glycerophospholipid pathway may bridge carbohydrate and fatty acid metabolism, potentially supporting growth and lipid homeostasis via *β*‐oxidation and adenosine triphosphate (ATP) production [71]. In addition, studies have shown that enrichment of glycerophospholipid metabolism suggests enhanced remodeling and resynthesis of membrane phospholipids, which may help repair damaged membrane structures and stabilize lipid droplet monolayers under high‐fat conditions [72]. In summary, LBP significantly modulated hepatic glycerophospholipid and nucleotide profiles while reducing bile acid metabolites. However, due to experimental constraints, the long‐term efficacy and optimal dosage of LBP in adult fish necessitate further investigation. Furthermore, while the observed correlations between gut microbiota and hepatic metabolites provide preliminary insights into the mechanisms of LBP, these findings remain speculative; future functional studies are essential to definitively establish a causal link within the gut–liver axis.

## 5. Conclusion

**Figure 12:**
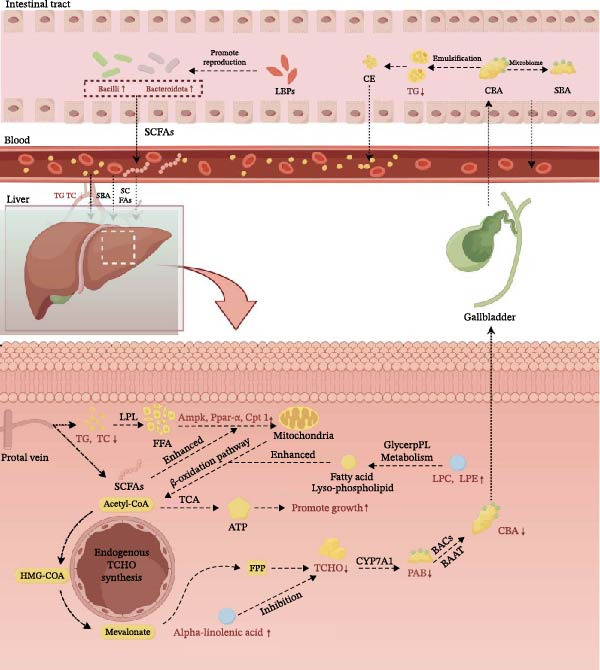
LBP alleviates lipid metabolism disorder in *T. obscurus*, speculating a regulatory role of the gut–liver axis. (By Figdraw). Note: Indicators shown in red represent variables that exhibited statistically significant differences in this experiment. The abbreviations in the figure are as follows: CBAs, conjugated bile acids; PBAs, primary bile acids; SBAs, secondary bile acids; TGs, triglycerides; FFAs, free fatty acids; CEs, cholesteryl esters; HMG‐CoA, 3‐hydroxy‐3‐methylglutaryl‐coenzyme A; FPP, farnesyl pyrophosphate; TCA, Tricarboxylic acid cycle; GlyceroPL Metabolism, glycerophospholipid metabolism; LPL, lipoprotein lipase; ATP, adenosine triphosphate; BACS, bile acid‐CoA synthetase; BAAT, bile acid‐CoA:amino acid N‐acyltransferase; SCFAs, short‐chain fatty acids.

Overall, this study demonstrates that LBPs can alleviate lipid metabolism disorders and enhance growth performance in *T. obscurus*. LBP appears to regulate gut microbiota‐mediated bile acid metabolism in the liver, thereby mitigating bile acid imbalance induced by a HFD (Figure 12). These findings provide a theoretical basis for the use of LBP as a green, safe, and antibiotic‐free functional feed additive and offer new insights into optimizing high‐fat feeding strategies and advancing health‐oriented aquaculture practices.

However, this study primarily focused on the potential mechanisms by which LBP is associated with the alleviation of lipid metabolism disorders in fish, and the relatively small sample size limits the generalizability of these findings. As such, this work should be considered a preliminary exploratory study, and larger‐scale experiments are required to validate the current results. Moreover, whether the lipid‐regulating effects of LBP are influenced by its specific structural features—such as monosaccharide composition and molecular weight—remains to be further elucidated. Additionally, while a high degree of correlation was observed between LBP‐induced shifts in the gut microbiota and hepatic differential metabolites, these findings remain associative in nature. Consequently, we speculate that the gut–liver axis may play a potential role in the regulation of lipid metabolism disorders in *T. obscurus*, a hypothesis that provides a theoretical basis for future investigations into the precise causal mechanisms.

## Author Contributions

Shenglin Yue and Fuqiang Wang designed the study. Shenglin Yue and Zixiang Lin conducted the experiments. Shenglin Yue, Zixiang Lin, Yiyang Huang, Tinghao Ma, Xiaoran Zhao, Yuzhe Han, Tongjun Ren, and Fuqiang Wang collected the samples. Shenglin Yue and Zixiang Lin analyzed the data. Shenglin Yue, Zixiang Lin, and Yiyang Huang wrote the manuscript and prepared the charts. All authors discussed the results together. Fuqiang Wang reviewed the manuscript draft. Fuqiang Wang and Xiuli Wang obtained financial support for the study.

## Funding

This work was supported by the Key Laboratory of Pufferfish Breeding and Culture in Liaoning Province (Grant 2021JH13/10200005), the Dalian Science and Technology Talent Innovation Support Policy Project (Grant 2024RG008), the Dalian Ocean University Science and Technology Innovation Team Project (Grant B202102), the Liaoning Province Applied Basic Research Program (Grant 2025JH2/101300086), and the Liaoning Provincial Major Science and Technology Project (Grant 2025JH1/11700001).

## Disclosure

All authors have read and approved this version of the manuscript and have taken the necessary precautions to ensure the integrity of the work.

## Conflicts of Interest

The authors declare no conflicts of interest.

## Supporting Information

Additional supporting information can be found online in the Supporting Information section.

## Supporting information


**Supporting Information 1** Table S1: List of standards used for LBP component analysis, including purity and retention times.

**Supporting Information 2** Figure S1: Total ion chromatograms (TICs) of monosaccharide analysis. (A) Monosaccharide standards. (B) Monosaccharides detected in LBP.

**Supporting Information 3** Figure S2: HPSEC‐MALLS‐RI chromatogram for molecular weight determination of LBP.

**Supporting Information 4** Figure S3: Molecular conformation plot of LBP.

**Supporting Information 5** Figure S4: Heatmap of differential microbiota at the phylum level among NFD, HFD, and HFD3 groups.

**Supporting Information 6** Figure S5: Heatmap of differential microbiota at the class level among NFD, HFD, and HFD3 groups.

**Supporting Information 7** Figure S6: Heatmap of differential metabolites between the HFD and NFD groups.

**Supporting Information 8** Figure S7: Heatmap of differential metabolites between the HFD3 and NFD groups.

**Supporting Information 9** Figure S8: Heatmap of differential metabolites between the HFD3 and HFD groups.

## Data Availability

Data will be made available upon request.
