# Supplementary material for: Lycium barbarum Polysaccharides Alleviate High‐Fat Diet–Induced Lipid Metabolism Disorder in Takifugu obscurus
Source: Aquac Nutr. 2026 Apr 29;2026:9289590. doi: 10.1155/anu/9289590 (PMC13126255; doi:10.1155/anu/9289590)
Supplement: Supplementary file 1 — Supporting Information 1 Table S1: List of standards used for LBP component analysis, including purity and retention times. [file ANU-2026-9289590-s001.docx]

| **No.** | **Compounds** | **Source** | **RT（min）** | **Peak Area** | **Purity** |
| --- | --- | --- | --- | --- | --- |
| 1 | Fuc | Yuanye | 5.1 | 12.477 | 99% |
| 2 | GalN | Yuanye | 10.109 | 18.855 | 98% |
| 3 | Rha | Yuanye | 10.584 | 14.829 | 98% |
| 4 | Ara | Yuanye | 11.7 | 18.164 | 98% |
| 5 | GlcN | Yuanye | 12.9 | 37.564 | 98% |
| 6 | Gal | Yuanye | 15.242 | 12.353 | 99% |
| 7 | Glc | Yuanye | 17.309 | 27.703 | 98% |
| 8 | Xyl | Yuanye | 20.367 | 25.511 | 99% |
| 9 | Man | Sigma-Aldrich | 21.184 | 16.884 | 99% |
| 10 | Fru | Yuanye | 24.642 | 8.645 | 98% |
| 11 | Rib | Yuanye | 27.275 | 21.595 | 99% |
| 12 | GalA | Yuanye | 42.542 | 14.031 | 97% |
| 13 | GulA | Bvant | 43.3 | 3.211 | 98% |
| 14 | GlcA | Yuanye | 45.075 | 25.423 | 98% |
| 15 | ManA | Yuanye | 47.284 | 10.523 | 99% |
