# Supplementary figures and images for: Lycium barbarum Polysaccharides Alleviate High‐Fat Diet–Induced Lipid Metabolism Disorder in Takifugu obscurus
Source: Aquac Nutr. 2026 Apr 29;2026:9289590. doi: 10.1155/anu/9289590 (PMC13126255; doi:10.1155/anu/9289590)

A

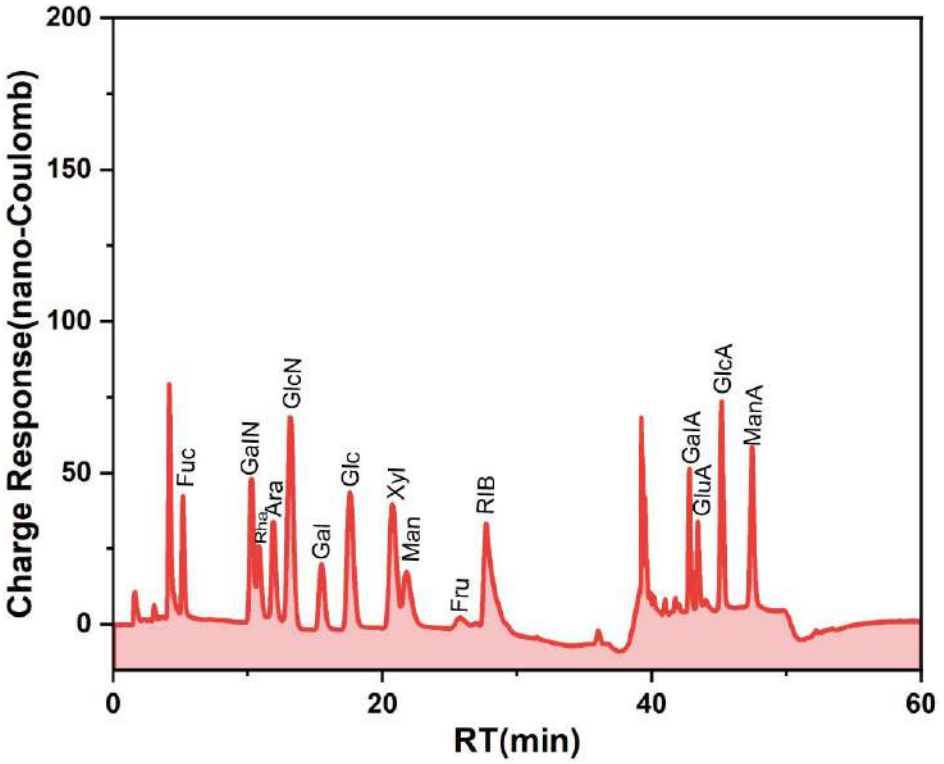

B

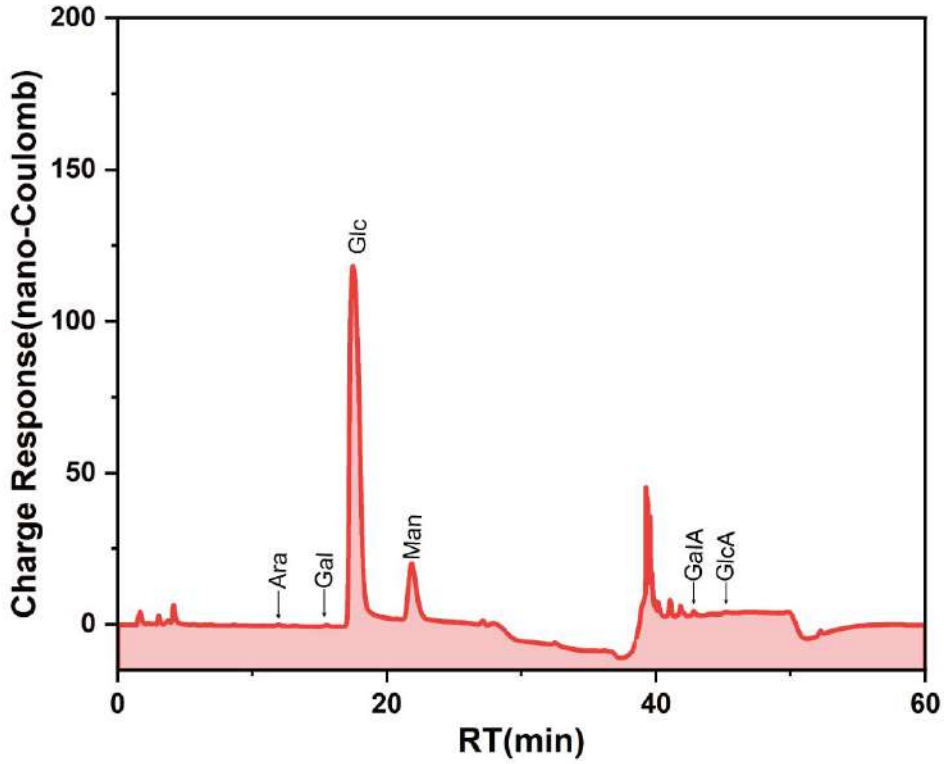

Supplement: Supplementary file 2 — Supporting Information 2 Figure S1: Total ion chromatograms (TICs) of monosaccharide analysis. (A) Monosaccharide standards. (B) Monosaccharides detected in LBP. [file ANU-2026-9289590-s002.pdf]

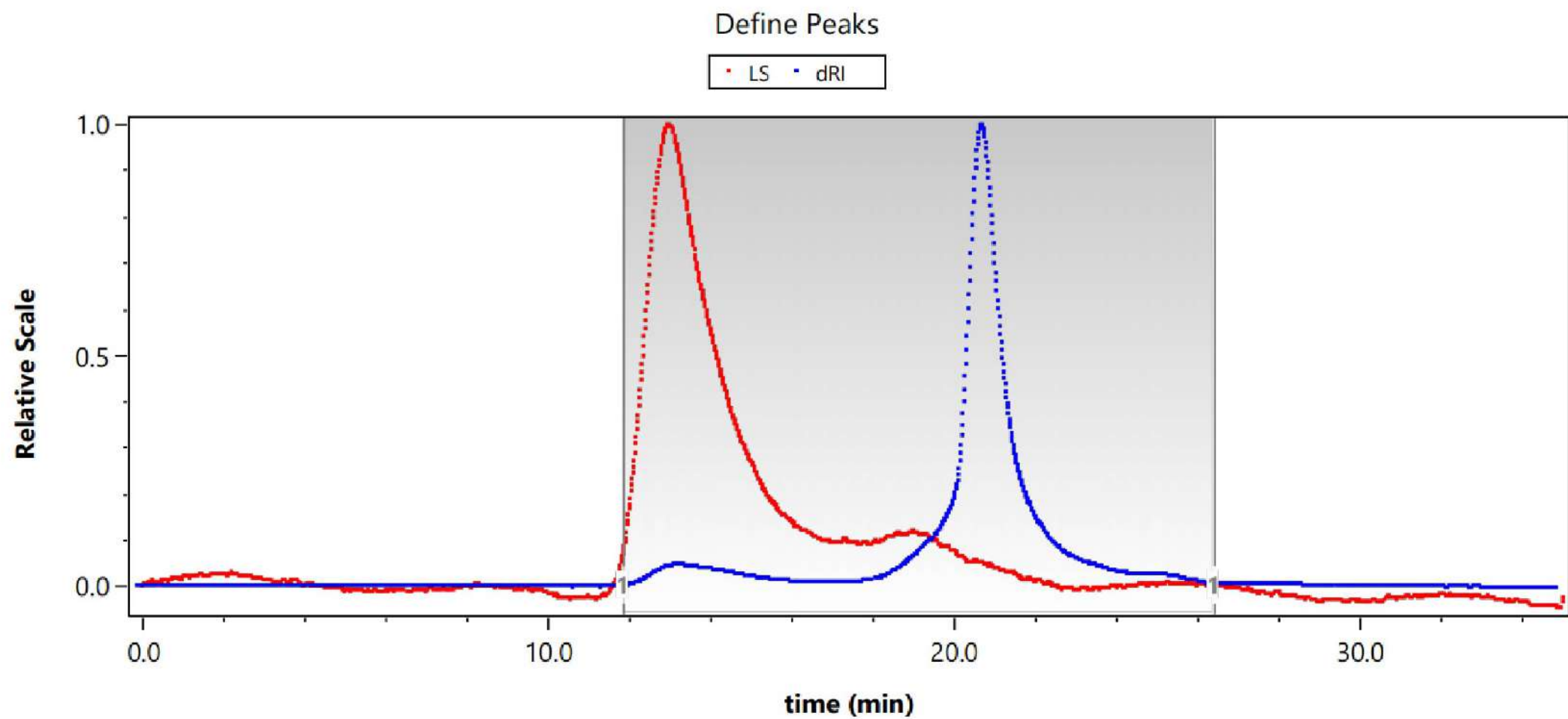

Supplement: Supplementary file 3 — Supporting Information 3 Figure S2: HPSEC‐MALLS‐RI chromatogram for molecular weight determination of LBP. [file ANU-2026-9289590-s003.pdf]

# Molar Mass vs. time

— GQDT[20250920]

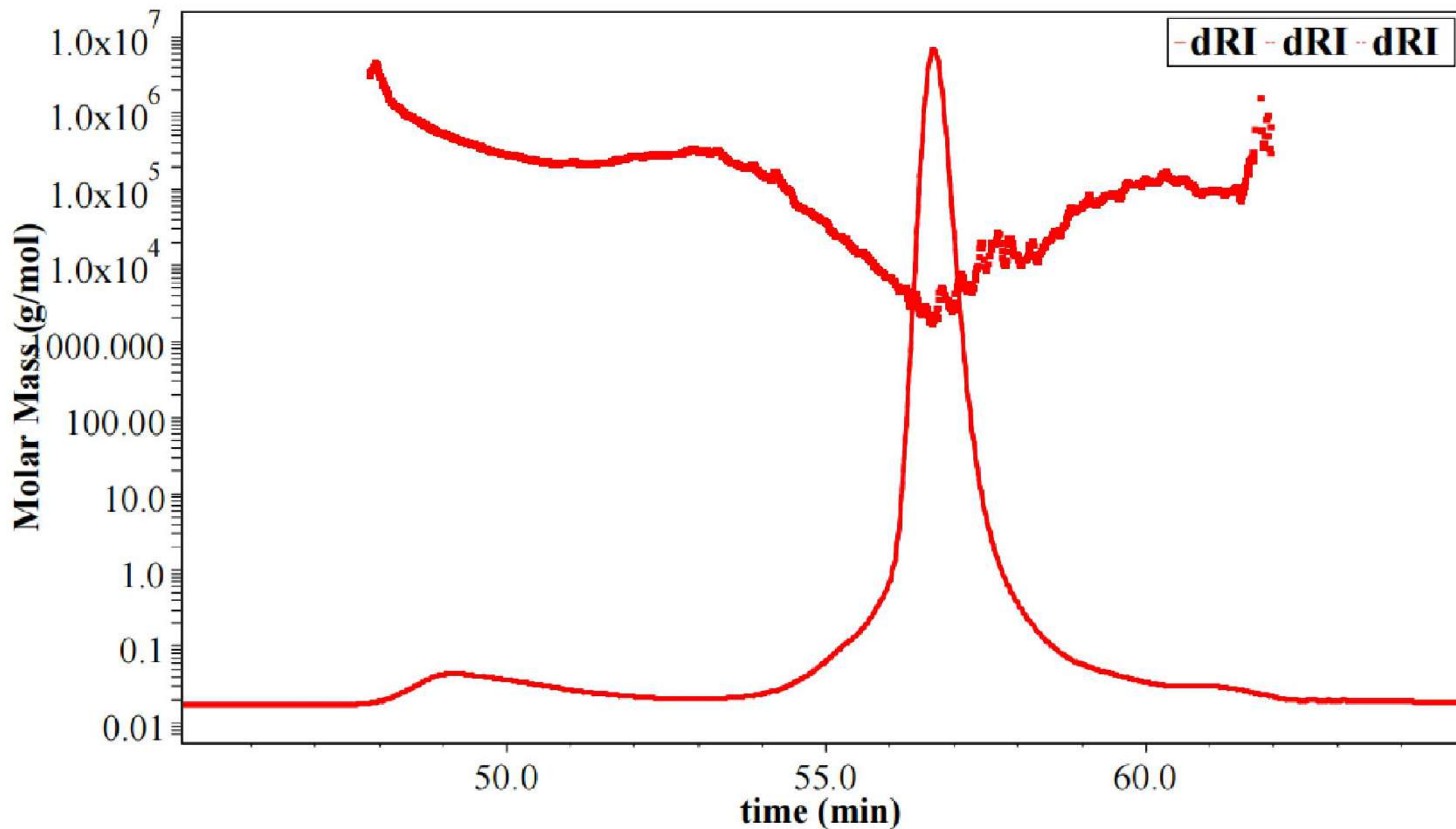

Supplement: Supplementary file 4 — Supporting Information 4 Figure S3: Molecular conformation plot of LBP. [file ANU-2026-9289590-s004.pdf]

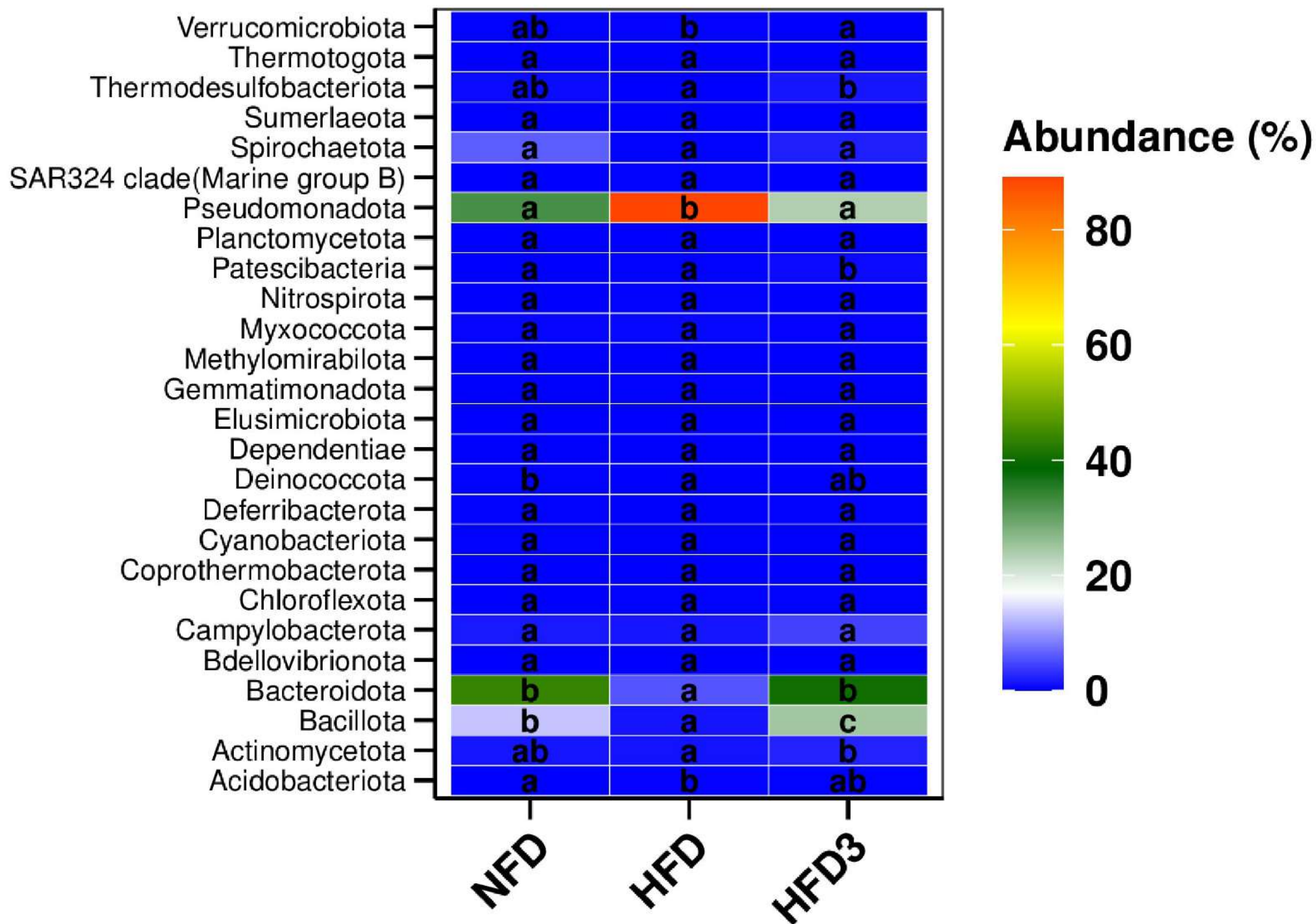

Supplement: Supplementary file 5 — Supporting Information 5 Figure S4: Heatmap of differential microbiota at the phylum level among NFD, HFD, and HFD3 groups. [file ANU-2026-9289590-s009.pdf]

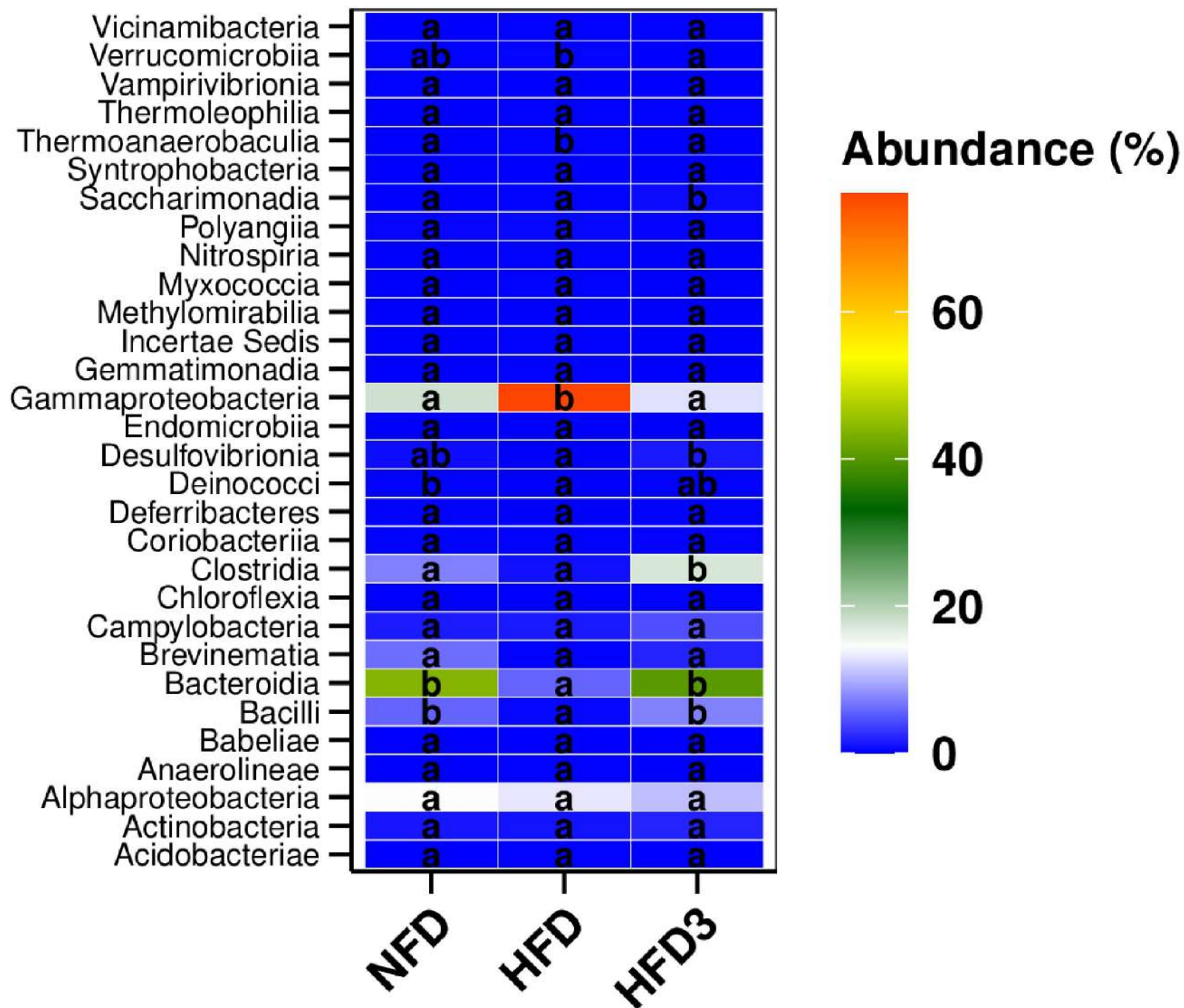

Supplement: Supplementary file 6 — Supporting Information 6 Figure S5: Heatmap of differential microbiota at the class level among NFD, HFD, and HFD3 groups. [file ANU-2026-9289590-s006.pdf]

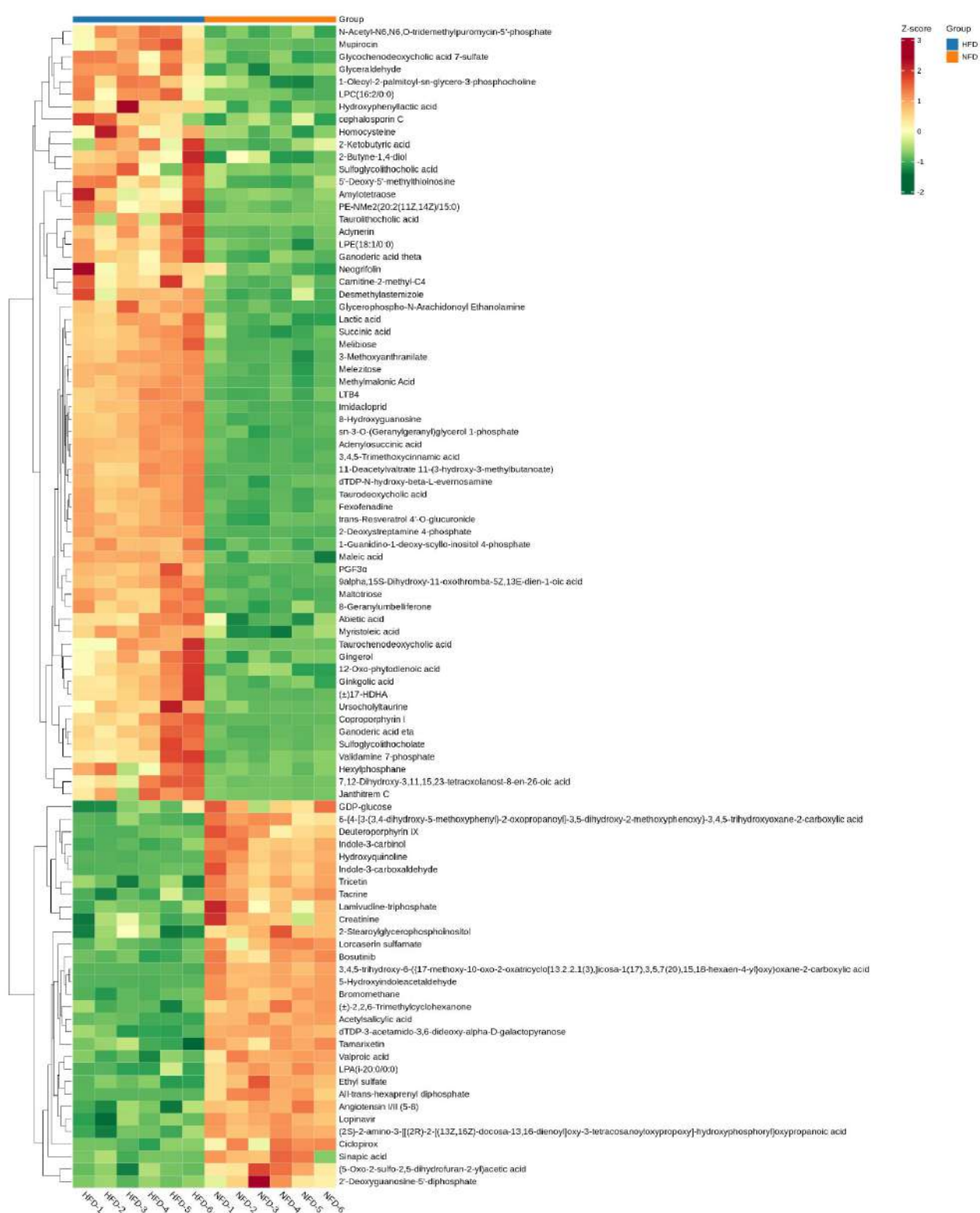

Supplement: Supplementary file 7 — Supporting Information 7 Figure S6: Heatmap of differential metabolites between the HFD and NFD groups. [file ANU-2026-9289590-s007.pdf]

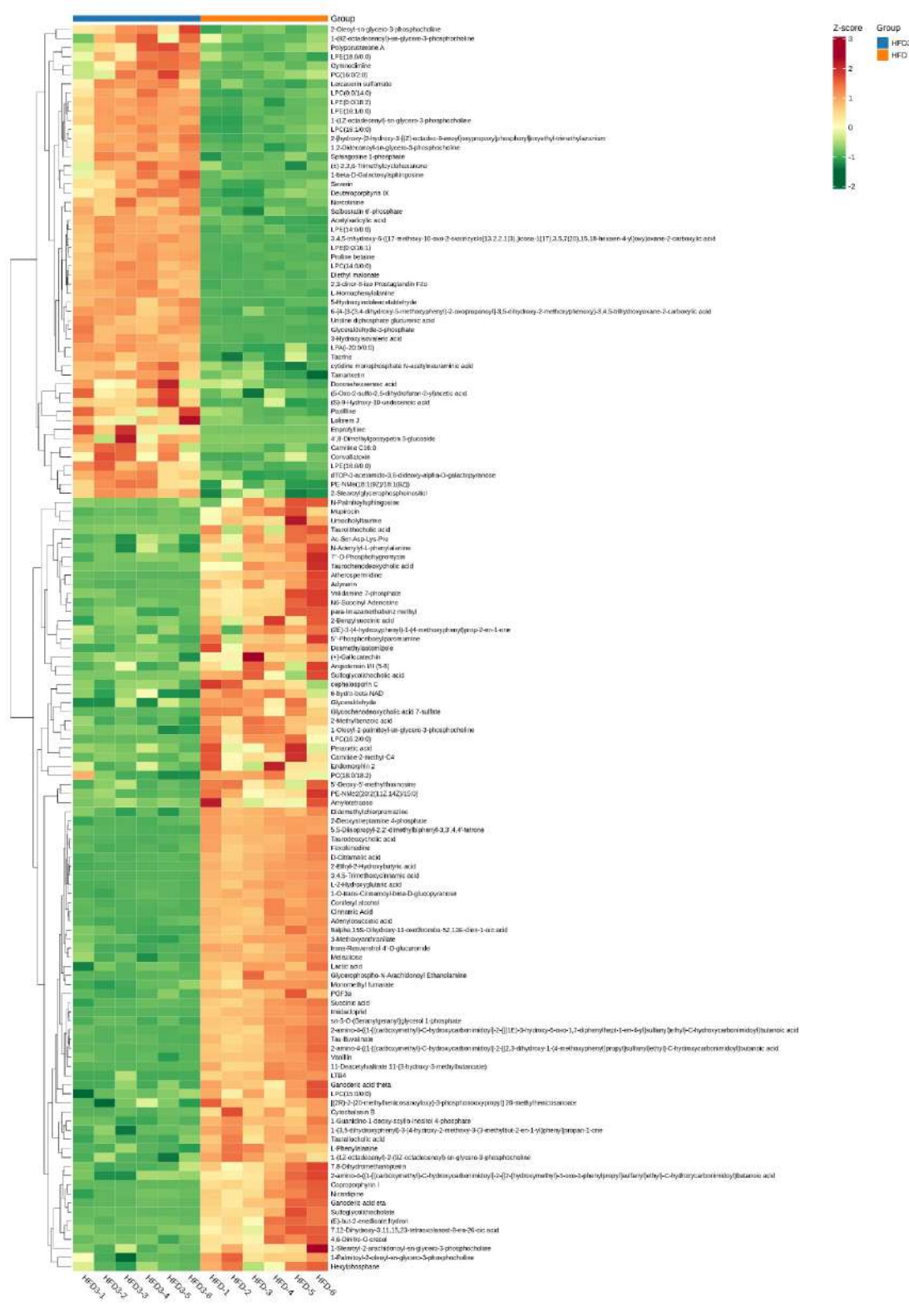

Supplement: Supplementary file 8 — Supporting Information 8 Figure S7: Heatmap of differential metabolites between the HFD3 and NFD groups. [file ANU-2026-9289590-s008.pdf]

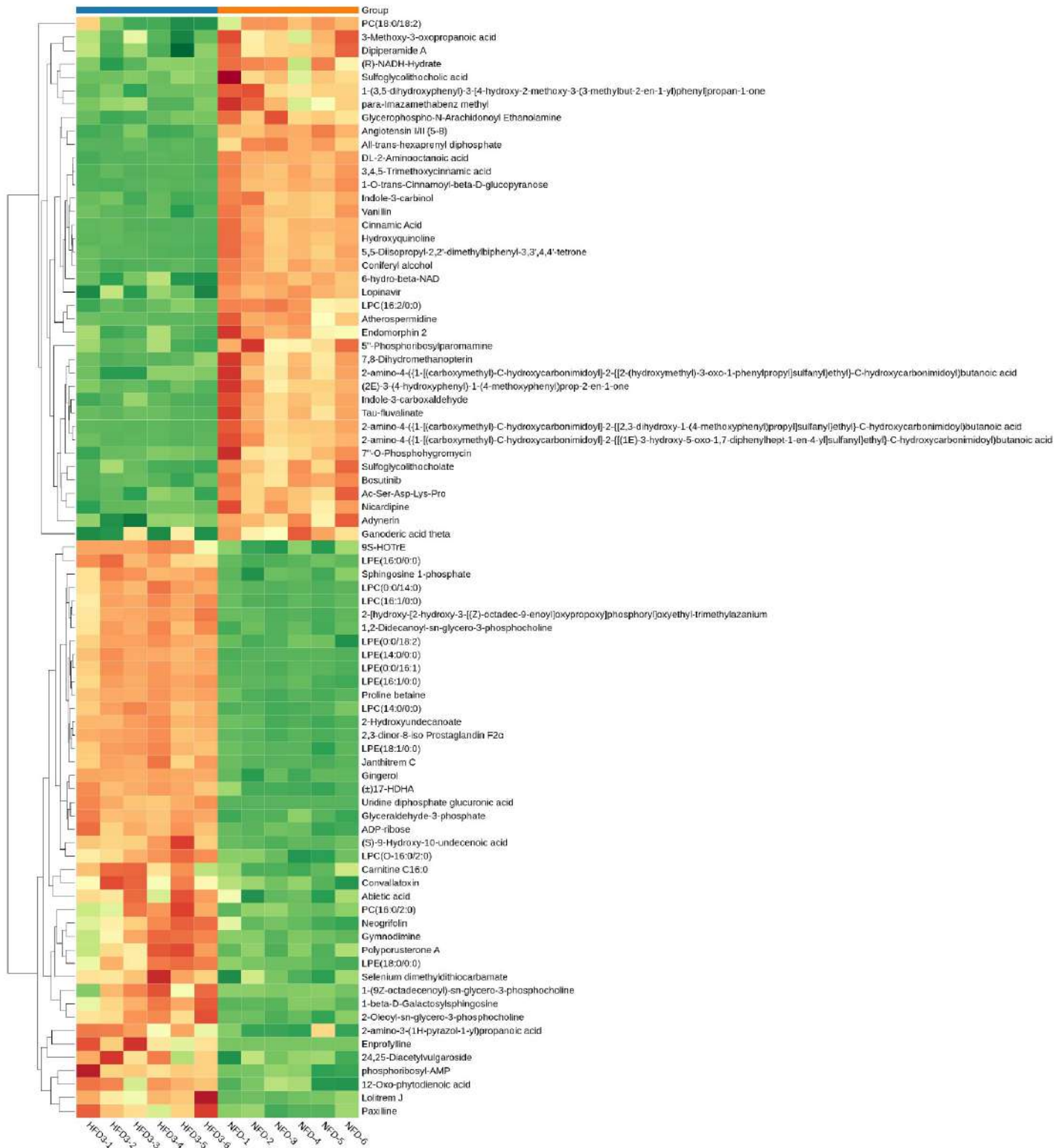

Supplement: Supplementary file 9 — Supporting Information 9 Figure S8: Heatmap of differential metabolites between the HFD3 and HFD groups. [file ANU-2026-9289590-s005.pdf]
